# A minimal RNA-cleaving DNAzyme and its catalytic mechanism

**DOI:** 10.1093/nar/gkaf1502

**Published:** 2026-01-15

**Authors:** Kazuhiko Yamasaki, Rika Inomata, Tomoko Yamasaki, Tomomi Kubota, Naoyuki Miyashita, Koh Takeuchi, Makoto Miyagishi

**Affiliations:** Molecular Biosystems Research Institute, National Institute of Advanced Industrial Science and Technology (AIST), 1-1-1 Higashi, Tsukuba 305-8566, Japan; Health and Medical Research Institute, National Institute of Advanced Industrial Science and Technology (AIST), 1-1-1 Higashi, Tsukuba 305-8566, Japan; Master’s/Doctoral Program in Life Science Innovation, School of Integrative and Global Majors, University of Tsukuba, 1-1-1 Tennodai, Tsukuba 305-8577, Japan; Molecular Biosystems Research Institute, National Institute of Advanced Industrial Science and Technology (AIST), 1-1-1 Higashi, Tsukuba 305-8566, Japan; Molecular Biosystems Research Institute, National Institute of Advanced Industrial Science and Technology (AIST), 1-1-1 Higashi, Tsukuba 305-8566, Japan; Faculty of Biology-Oriented Science and Technology, KINDAI University, 930 Nishimitani, Kinokawa, Wakayama 649-6493, Japan; Graduate School of Pharmaceutical Sciences, The University of Tokyo, 7-3-1 Hongo, Bunkyo-ku, Tokyo 113-0033, Japan; Health and Medical Research Institute, National Institute of Advanced Industrial Science and Technology (AIST), 1-1-1 Higashi, Tsukuba 305-8566, Japan; Master’s/Doctoral Program in Life Science Innovation, School of Integrative and Global Majors, University of Tsukuba, 1-1-1 Tennodai, Tsukuba 305-8577, Japan

## Abstract

Although natural sources of enzymes are limited to protein and RNA, some artificial DNAs exhibit catalytic activities. Representative functions of such DNAs, i.e. DNAzymes, are cleavage and ligation of nucleic acids. Here we developed a minimal DNAzyme with an RNA-cleaving activity by *in vitro* selection and secondary structure-based design. Its catalytic and substrate cores are only two and three nucleotides, respectively. This DNAzyme showed strict Zn^2+^ dependence at optimal pH 7.0–7.5. To elucidate its catalytic mechanism, we determined its three-dimensional structure by X-ray crystallography and nuclear magnetic resonance (NMR) spectroscopy. The results consistently showed a B-DNA-like structure with base pairing and stacking throughout the molecule, unlike the kinked structures of larger DNAzymes. Notably, an A-base in the catalytic loop and a G-base in the substrate loop formed a non-Watson–Crick base pair. The catalytic Zn^2+^ coordinates to N7 of that G-base, enabling the Zn^2+^-hydrated water molecules to contacts O2′ and O5′ at the cleavage site. Considering that Zn(OH)^+^ and Zn^2+^ co-exist at the enzyme’s optimal pH, we propose a catalytic mechanism whereby these ions act as the base withdrawing H^+^ from O2′ and the acid donating H^+^ to O5′, generating the cleaved ends with 2′,3′-cyclic phosphate and OH groups.

## Graphical Abstract

**Graphical Abstract:**
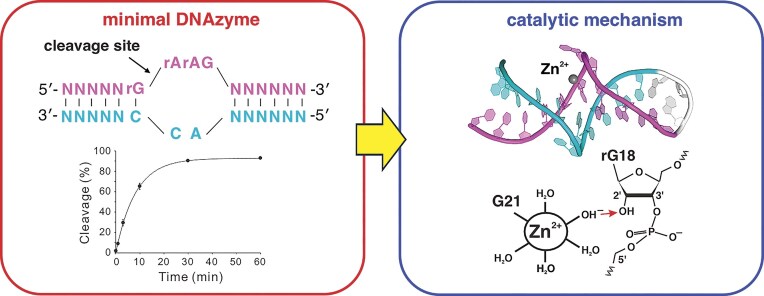

## Introduction

Natural sources of enzymes are restricted to protein and RNA, although some DNAs originating from *in vitro* screening of artificial DNA libraries exhibit catalytic activities [1]. Such DNAs, namely DNAzymes, catalyze diverse reactions, including the cleavage and ligation of RNA or DNA [1–6], phosphorylation [7], and metalation of porphyrin [8]. Due to the chemical and physiological stability of DNA compared with RNA, extensive research has been focused on the utilization of DNAzymes in biosensors and antiviral or anticancer therapeutics [9–13]. RNA-cleaving DNAzymes, in particular, are considered highly promising because they can target the mRNAs of virtually any gene by designing complementary hybridization sequences.

Like ribozymes, most DNAzymes require divalent cations, among which Zn^2+^ pre-exists in cells and plays biologically important roles. Specifically, it forms the structural core of a number of proteins, such as zinc finger transcription factors in which Cys and His residues coordinate with Zn^2+^ [14]. The first Zn^2+^-dependent DNAzyme was selected on the basis of its RNA-cleaving activity [3]. This 17E DNAzyme showed a sequence motif highly related to RNA-cleaving 8-17 DNAzyme [2], which was previously selected under conditions involving Mg^2+^ ions. It was shown, however, that these 8-17-related DNAzymes are much more active with Pb^2+^ and Zn^2+^ than with Mg^2+^, regardless of the original selection schemes [15].

Three-dimensional (3D) structures provide critical information on the catalytic mechanism involving the metal ions. Several crystal and solution structures of DNAzymes, such as 9DB1 [16], 8-17 [17, 18], and 10-23 [19–21], are currently available, where the former has RNA-ligating activity and the latter two have RNA-cleaving activity. It should be noted that the crystal [19, 21] and solution [20] structures of 10-23 DNAzyme significantly differ from each other, where the former formed artificial homodimers probably due to the crystal packing. In contrast, crystal [17] and solution [18] structures of 8-17 DNAzymes are essentially identical. Some of these structures include catalytic metals such as Pb^2+^, whereas no Zn^2+^-containing DNAzyme structures have been reported. Although the solution structure of the 8-17 DNAzyme was determined in the presence of Zn^2+^, the ion was not visible by nuclear magnetic resonance (NMR). For ribozymes, a Zn^2+^-containing structure of a mutant of the hammerhead ribozyme was reported, where a Zn^2+^ ion coordinating to the N7 atom of a G base was considered to participate directly in the cleavage reaction [22].

Here we aim to obtain a small *trans*-acting Zn^2+^-dependent DNAzyme that cleaves RNA. Our selection scheme primarily followed the method used for short *trans*-acting ribozymes, where the enzyme strand is released from the immobilized substrate strand if the latter is successfully cleaved [23]. The nucleic acids for the library and substrate we used were derived from a Zn^2+^-dependent DNA-cleaving DNAzyme, I-R3 [5]. This is because I-R3 has a compact catalytic core of 10 bases and showed better cleavage efficiency than other DNA-cleaving DNAzymes. According to the obtained sequence, we could further reduce the size of the DNAzyme to only two and three nucleotides in the catalytic and substrate core, respectively. Considering the merits and defects in X-ray crystallography and NMR spectroscopy, as described above, we used both methods to determine its 3D structure in crystal form and in solution, which revealed a stereochemical basis for the Zn^2+^-dependent RNA-cleaving reaction.

## Materials and methods

### *In vitro* selection

The N10-DNA library and the corresponding substrate strands (N10-DNA and sub-N10 in Supplementary Table S1) were synthesized by Hokkaido System Science Co., Ltd (Sapporo, Japan). Polymerase chain reaction (PCR) primers (N10-DNA-F and N10-DNA-R in Supplementary Table S1) were supplied by Eurofins Genomics (Tokyo, Japan).

Selection experiments were performed basically using the methods reported by Conaty *et al.* [23] and Fukuda *et al.* [24] The 3′-biotinylated substrate strand (200 pmol) was incubated with Dynabeads™ MyOne™ Streptavidin C1 beads (Thermo Fisher Scientific Inc., Waltham, MA, USA) in a wash buffer [50 mM HEPES (pH 7.5), 1 M NaCl, and 0.01% Tween 20] for 1 h at room temperature. After washing three times with the above wash buffer, the N10-DNA library (200 pmol) was annealed to the substrate strand by heating at 95°C for 5 min and gradually cooling to 4°C. After washing out unbound strands, the library–substrate complexes on beads were incubated in the reaction buffer [50 mM HEPES (pH 7.5), 150 mM NaCl, 1 mM ZnCl_2_, and 0.01% Tween 20] at 37°C for 30 min to elute the library sequences which have an RNA cleavage ability. Three rounds of selection were performed, in which the eluted library obtained from each round was directly used for the next round without amplification. Following the third round of selection, the selected library was amplified by PCR using Takara Ex Taq (TaKaRa, Shiga, Japan). In some cases, the amplified DNA was purified by acrylamide gel electrophoresis. The resulting double-stranded DNA sequences were further amplified using primers containing barcode sequences for next-generation sequencing. Deep sequencing was carried out by Illumina MiSeq (Illumina Inc., San Diego, CA, USA) using a MiSeq Reagent Kit V2 (Illumina). The ~30 000 read sequences obtained were analyzed using the MEME Suite 5.0.1 motif analysis tool (http://meme-suite.org/tools/meme) [25]. The top 523 sequences were used to identify enriched motifs. The candidate library sequences in the motifs were chemically synthesized and used for experiments in a substrate cleavage assay.

To elucidate requirements in the catalytic core sequence, an *in vitro* selection experiment using the N3-DNA library, corresponding substrate DNA (N3-DNA and sub-N3 in Supplementary Table S1; Hokkaido System Science), and primers (N3-DNA-F and N3-DNA-R; Eurofins) was also performed essentially in the same manner as above.

### Designed DNAzymes for minimization

Designed DNAzymes (IR3-R, IR3-R-wo4, and minGAA in Supplementary Table S1), a negative control (IR3 in Supplementary Table S1), and the substrate strand (sub-rArAG in Supplementary Table S1) were purchased from Eurofins Genomics. Variants with regard to the second bulge and the arm length (minGAA-iACGT, minGAA-iGATC, minGAA-s, and minGAA-77 in Supplementary Table S1) were purchased from Hokkaido System Science (some batches of minGAA-s were obtained from Eurofins). These DNAzymes and substrate were incubated in the reaction buffer [50 mM HEPES (pH 7.5), 150 mM NaCl, and 1 mM ZnCl_2_] under single turnover conditions (DNAzyme:substrate = 10:1; 1 μM DNAzyme) to compare the percentage cleavage by electrophoresis on a denaturing gel (20% polyacrylamide/bis and 7 M urea). The cleavage products were quantified from the gel images obtained using a ChemiDoc™ XRS+ imaging system (Bio-Rad, Hercules, CA, USA). The average and the standard deviation (SD) were calculated from three parallel experiments. Kinetic parameters were obtained as described in the Supplementary Methods. In addition, kinetic experiments under multiple turnover conditions (DNAzyme:substrate = 1:5, 1:10, 1:20, 1:40, and 1:80; 0.1 μM DNAzyme) were performed for minGAA-s and minGAA-77.

### Zn^2+^ concentration dependency and pH profile and metal selectivity of minGAA

The Zn^2+^ concentration dependence and pH profile were investigated under single turnover conditions (minGAA-s:substrate = 10:1). The DNAzymes were annealed to the FAM substrate and the cleavage reactions were compared under each buffer condition. For the pH profile, 50 mM HEPES with different pH values (6.0, 6.5, 7.0, 7.5, and 8.0) were used in a reaction buffer (150 mM NaCl and 1 mM ZnCl_2_), where kinetic experiments were also performed, as shown in the Supplementary Methods. For Zn^2+^ concentration dependence, the experiments were carried out at 37°C for 1 h in a reaction buffer [50 mM HEPES (pH 7.5) and 150 mM NaCl] with different zinc concentrations (ZnCl_2_ at final concentrations of 0, 0.1, 0.3, 1, 3, or 10 mM).

To elucidate the metal ion selectivity, activities were examined under single turnover conditions in the presence of divalent metal ions (0.1, 1, or 10 mM MgCl_2_, CaCl_2_, MnCl_2_, CoCl_2_, ZnCl_2_, and BaCl_2_) or monovalent metal ions (1 M LiCl, NaCl, and KCl). The enzyme strand and substrate strand were annealed in buffer [50 mM HEPES (pH 7.5) and 150 mM NaCl]. To start the reaction, metal ions were added into the buffer and the samples were incubated at 37°C for 30 min. The cleavage activities were determined by gel electrophoresis and following gel imaging as described above. Kinetic experiments of minGAA-s in the above buffer containing 1 mM ZnCl_2_ and 10 mM MgCl_2_ were also performed.

### Elucidation of mutations in the catalytic core and substrate sequence specificity

The 15 variants of minGAA-s DNAzyme (minGAA-AA, etc., in Supplementary Table S1) were chemically synthesized by Hokkaido System Science Co., Ltd and examined under single turnover conditions. The cleavage reactions were carried out in buffer [50 mM HEPES (pH 7.5), 1 mM ZnCl_2_, and 150 mM NaCl] at 37°C for 1 h.

The 3′-FAM-substrate strands, having different core sequences in the RNA region (sub-rCrAG, sub-rArCG, and sub-rArAC in Supplementary Table S1), were synthesized by Eurofins. The reactions were performed under single turnover conditions in reaction buffer [50 mM HEPES (pH 7.5), 150 mM NaCl, and 1 mM ZnCl_2_] at 37°C for 1 h to compare the cleavage activities by minGAA for each substrate. In addition, all-RNA substrate, single-RNA substrate, and variants at the cleavage site rG (sub-allRNA, sub-singleRNA, sub-rArArAG, sub-rCrArAG, and sub-UrArAG in Supplementary Table S1; Hokkaido System Science) were subjected to the kinetic experiments with minGAA-s.

### Analysis of substrate sequence specificity by *in vitro* selection

The N3 library with a 3 nt randomized RNA region (rN3-Sub in Supplementary Table S1; Hokkaido System Science) were annealed with minGAA-s. The reactions were performed under single turnover conditions (minGAA-s:rN3-Sub library = 10:1) in reaction buffer [50 mM HEPES (pH 7.5) and 150 mM NaCl] with or without 1 mM ZnCl_2_ at 37°C for 30 min. After an ethanol precipitation to remove the metal ions and PCR amplification with primers (rN3-Sub-F and rN3-Sub-R in Supplementary Table S1), sequences of the uncleaved molecules were read by the next-generation sequencer and the numbers of sequences were counted. The efficiencies of the cleavage were evaluated by the ratios of the numbers of the respective sequences obtained in the presence or absence of ZnCl_2_.

### Liquid chromatography–mass spectrometry

A 4 µM concentration of *cis*-type minGAA (*cis*-minGAA in Supplementary Table S1; Eurofins) was incubated in buffer [50 mM HEPES (pH 7.5), 1 mM ZnCl_2_, and 150 mM NaCl] at 37°C for 1 h. After purification of the cleaved nucleic acids by ethanol precipitation, liquid chromatography–mass spectrometry (LC-MS) was performed by Gene Design Inc. (Osaka, Japan).

### Crystallography

Two types of nucleic acid molecules—type 1 (*cis*-minGAA-type-1 in Supplementary Table S1: equivalent to the above *cis*-type minGAA) and type 2 (minGAA-s-type-2/sub-type-2 in Supplementary Table S1)—were purchased from Hokkaido System Science. Details of crystallography are described in the Supplementary Methods. The coordinates and structure factor data were deposited in the Protein Data Bank (PDB) under accession IDs 9k8p (type 1) and 9k8o (type 2).

### NMR analyses

Nucleic acid molecules used for NMR analysis were type 1, the same as above, and its derivatives dA19 and dA20 (*cis*-minGAA-type-1-dA19 and *cis*-minGAA--1-dA20 in Supplementary Table S1, respectively), which were purchased from Hokkaido System Science. Details of NMR analyses including calculation methods are described in the Supplementary Methods.

Coordinates and chemical shift values were deposited in the PDB and the Biological Magnetic Resonance Bank (BMRB) under accession IDs 9k8n and 36699.

### Molecular dynamics simulations

Two molecular dynamics (MD) simulations were conducted. The first simulation employed the preliminary Zn^2+^-free NMR structure of minGAA as the initial configuration in an aqueous environment containing 150 mM Na^+^ and Cl^−^ ions (no Zn^2+^ ion). This system included 61 427 atoms. Our simulation protocol involved a 1000-step steepest descent minimization followed by 4 ns of equilibration calculation using GROMACS version 5.1 [26] with the AMBER14SB-parmbsc1 [27] force field. We then conducted a 2 μs MD simulation under the NPT ensemble, which has a temperature at 300 K and a pressure at 1 atm.

The second simulation employed the same minGAA structure as above and one Zn^2+^ ion. The initial position of the Zn^2+^ ion was set at the location where Na^+^ ions were most densely concentrated in the first simulation (Supplementary Fig. S11), given the unknown binding site. With the same environmental conditions as above, the system contained 61 683 atoms. We conducted minimization, equilibration, and a 2 μs MD simulation, also as above.

## Results and discussion

### Identification of the minimal Zn^2+^-dependent DNAzyme, minGAA

We set up an *in vitro* screening scheme to obtain nucleic acid enzymes, modeled after the scheme used for the DNA-cleaving DNAzyme I-R3 [5]. Specifically, we used a DNA library with a catalytic core DNA sequence of 10 randomized bases and a core substrate of 7 bases. Our screening successfully enriched the I-R3 sequence, allowing us to determine the optimal conditions for comprehensively and efficiently enriching I-R3-like sequence motifs.

**Figure 1.**
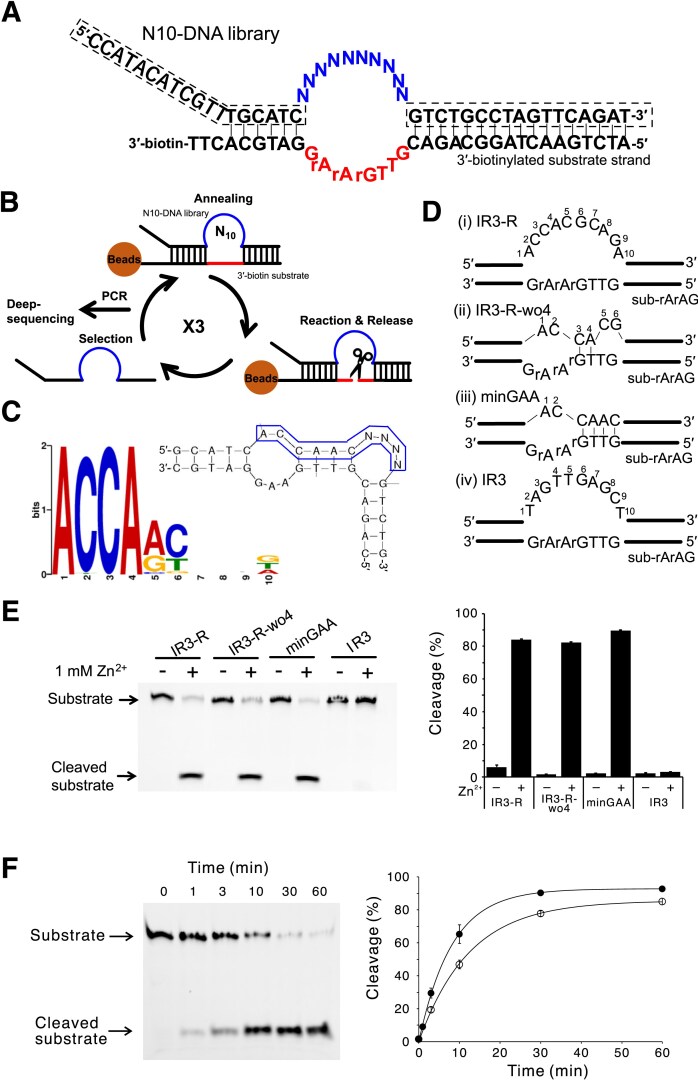
RNA-cleaving DNAzymes obtained by *in vitro* selection. (**A**) The sequences of the N10-DNA library and the 3′-biotinylated substrate used in the *in vitro* selection experiment. The 10 nt randomized sequence in the library is represented as Ns. The arm sequences of the DNA library, basically corresponding to primer sequences for amplification, are indicated with a dotted line. The catalytic core region and an assumed core substrate region are shown in blue and red, respectively. (**B**) The *in vitro* selection procedure used in this study. In each cycle, the DNA library sequences that exhibited cleavage activity were released into the solution. The catalytic core region and the core substrate RNA region are shown in blue and red, respectively. (**C**) A consensus sequence enriched by *in vitro* selection (left) and a predicted secondary structure of the candidate DNAzyme having the motif (5′-ACCAACNNNN-3′; right). (**D**) The designed DNAzymes used in the minimization experiment. (i) IR3-R: the most enriched sequence in the *in vitro* selection. (ii) IR3-R-wo4: a shortened DNAzyme derived by removing the seventh to 10th nucleotides of the catalytic core of IR3-R. (iii) minGAA: the minimized DNAzyme designed based on the MEME motif to form a duplex between the third and sixth nucleotides in the catalytic core and that of the substrate. (iv) The reported DNA-cleaving I-R3 DNAzyme [5]. (**E**) The 3′-FAM-labeled substrates were treated by IR3-R, IR3-R-wo4, minGAA, and IR3 with or without Zn^2+^ and analyzed by denaturing polyacrylamide gel electrophoresis (PAGE). The error bars in the right panel represent the SD from the average of three independent experiments at each point. (**F**) Kinetic experiment for minGAA molecules containing 9 + 21 and 9 + 9 nucleotide arms (filled and open circles, for minGAA and minGAA-s, respectively) under single turnover conditions.

Based on this screening system, we aimed to obtain novel RNA-cleaving DNAzymes and conducted the following screening experiments. Specifically, we designed a DNA library with a 10 nt randomized sequence (N10-DNA library) and the same substrate sequence as that for I-R3 except that the three DNA bases of the core sequence were replaced with RNA (5′-rGrArA-3′: “r” denotes a ribonucleotide; otherwise a deoxyribonucleotide, hereafter; Fig. 1A). The N10-DNA library was annealed with the 3′-biotinylated substrate strand bound to streptavidin-coated magnetic beads (Fig. 1B). After washing out unannealed and non-specifically bound molecules in the DNA library, the complexes of the two strands were incubated in a reaction buffer containing 1 mM ZnCl_2_ at 37°C for 30 min. During this period, DNAs with RNA-cleaving activity should be released into the supernatant. Such DNAs were recovered and again annealed with the substrate strand for repeating the selection scheme. After three cycles of the scheme, the recovered active DNAs were amplified by PCR with a primer set corresponding to the fixed-arm sequences of the N10-DNA library. Then the amplified sample was analyzed by next-generation sequencing, and ~30 000 read sequences were determined.

Analysis of the top 523 sequences with the highest appearance rate using MEME software [25] revealed an enrichment of a major sequence motif consisting of 453 sequences (Fig. 1C, left). The secondary structure of the candidate and substrate sequence complex was predicted by using the mfold software [28] to fold with two bulges surrounded by double strands (Fig. 1C, right). It was suggested that the second bulge (indicated by NNNN) does not contribute to the catalytic activity due to the lack of sequence consensus. This observation enabled us to design simple DNAzymes to cleave the substrate including the 5′-rGrArA-3′ sequence (Fig. 1D). The first designed DNAzyme termed IR3-R is the most frequent sequence in the *in vitro* selection; it has a 10 nt catalytic core sequence, recognizing a 7 nt substrate core sequence. The second DNAzyme (IR3-R-wo4) has a shorter sequence, which has a 6 nt catalytic core sequence formed by deleting four bases from the end of IR3-R, corresponding to the second bulge. The third DNAzyme (minGAA) contains a minimal sequence with only a 2 nt catalytic core sequence, produced by modification of two bases of IR3-R-wo4 in order that the nucleotides 3–6 in the catalytic core form a duplex. Consequently, the bulged region in the substrate strand consisted of only three nucleotides (5′-rArAG-3′). An *in vitro* cleavage experiment under single turnover conditions showed that the three designed DNAzymes, but not the original I-R3 enzyme, effectively cleaved the substrate sequence that includes 5′-rGrArA-3′ (Fig. 1E). Notably, minGAA is very compact, although its cleavage activity appears to be highest among the three designed DNAzymes.

Kinetic experiments were performed by monitoring the time dependence of the fraction cleaved for this minGAA molecule (containing 9 + 21 arm regions flanking the catalytic core) and for a further shortened variant (9 + 9 arms; minGAA-s) (Fig. 1F; Table 1). The latter showed slightly reduced activity, with a *k*_obs_ value of 0.081 min^−1^, compared with 0.123 min^−1^ for the former. Also, enzymes containing ACGT and GATC sequences in the second bulge shown in Fig .1C (minGAA-iACGT and minGAA-iGATC) exhibited slightly reduced activity compared with the bulge-deleted form (minGAA) (Supplementary Fig. S1; Table 1). These results confirmed that this second bulge did not contribute to catalysis. In addition, the minimization intermediates (IR3-R and IR3-R-wo4) also showed slightly lower activities (Supplementary Fig. S1; Table 1), consistent with the cleavage yields shown in Fig. 1E. Because minGAA and minGAA-s showed no substantial difference in kinetic activity, we used the latter, shorter molecule for the subsequent characterization experiments, where “minGAA” molecule refers to minGAA-s, unless otherwise stated.

**Table 1.** Kinetic parameters for cleavage reactions of various enzymes and substrates

Enzyme^a^	Substrate^a^	*k*_obs_ (min^–1^)^b^	Amplitude (%)^b^
minGAA	sub-rArAG	0.123 ± 0.012	92.9 ± 0.7
minGAA-iACGT	sub-rArAG	0.080 ± 0.004	83.4 ± 1.2
minGAA-iGATC	sub-rArAG	0.045 ± 0.002	82.2 ± 1.3
IR3-R	sub-rArAG	0.086 ± 0.001	88.4 ± 0.4
IR3-R-wo4	sub-rArAG	0.049 ± 0.005	89.6 ± 3.4
minGAA-s	sub-rArAG	0.081 ± 0.005	85.6 ± 1.6
minGAA-s	sub-allRNA	0.0118 ± 0.0004	90.0 ± 1.2
minGAA-s	sub-singleRNA	0.0040 ± 0.0002	(85.0)^c^
minGAA-s	sub-rArArAG	0.0023 ± 0.0001	(85.0)
minGAA-s	sub-rCrArAG	0.00078 ± 0.00025	(85.0)
minGAA-s	sub-UrArAG	0.00037 ± 0.00002	(85.0)

^a^Sequences are shown in Supplementary Table S1.

^b^Average values and SDs were obtained by bootstrap fitting of triplicate experiments (see the Supplementary Methods).

^c^Fixed during fitting calculations.

### Enzymatic properties of minGAA

**Figure 2.**
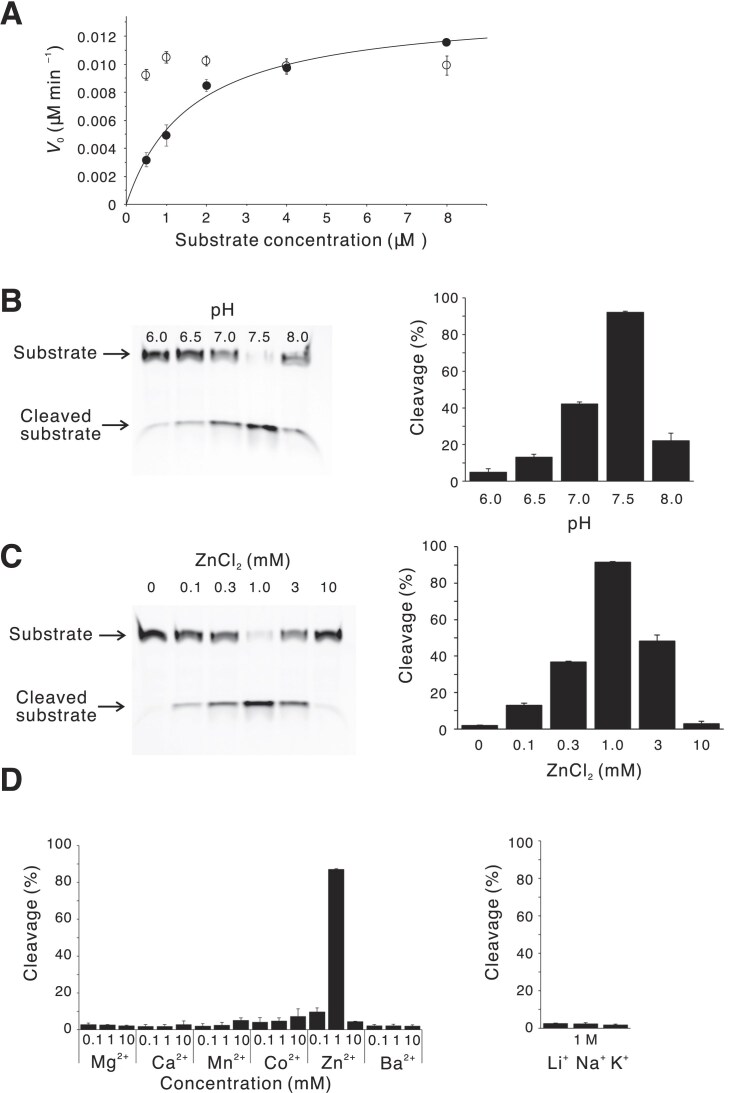
Enzymatic properties of minGAA DNAzyme. (**A**) Initial velocities obtained by a kinetic experiment for minGAA molecules containing 7 + 7 and 9 + 9 nucleotide arms (filled and open circles, for minGAA-77 and minGAA-s, respectively) under multiple turnover conditions. The curve fitted to the Michaelis–Menten equation is shown for minGAA-77. (**B**) pH and (**C**) Zinc concentration dependencies of the enzymatic activity. (**D**) The divalent metal ion selectivity (Mg^2+^, Ca^2+^, Mn^2+^, Co^2+^, Zn^2+^, and Ba^2+^) and the monovalent metal ion selectivity (Li^+^, Na^+^, and K^+^) of minGAA.

To obtain *k*_cat_ and *K*_m_ values, we evaluated initial velocities under multiple turnover conditions for minGAA-s with the 9 + 9 nucleotide arm region, which showed little dependence on the substrate concentration (Fig. 2A). In contrast, minGAA-77, containing a 7 + 7 nucleotide arm region, exhibited a clear substrate concentration dependence, which could be fitted to the Michaelis–Menten equation. The resulting *k*_cat_ and *K*_m_ values were 0.14 ± 0.01 min^−1^ and 1.6 ± 0.2 μM, respectively. These results suggest that the arm region must be sufficiently short to allow rapid product dissociation, which is essential for multiple turnover. We also suppose that *K*_m_, reflecting substrate affinity, is strongly influenced by the arm length, whereas *k*_cat_ is not.

We compared the *k*_cat_ and *K*_m_ values of minGAA with those of other DNAzymes, such as 10-23 and 8-17. The *k*_cat_ value is strongly dependent on the Mg^2+^ concentration for 10-23, and ranges from 0.15–0.49 min^–1^ at lower concentrations (2–10 mM MgCl_2_) to 1.7–3.4 min^–1^ at higher concentrations (50–100 mM Mg^2+^) [2, 29]. For 8-17, *k*_cat_ values of 0.01–0.67 min^–1^ have been reported under various divalent cations and temperatures [30]. Therefore, the *k*_cat_ value of minGAA is comparable with the lower end of the ranges reported for these well-characterized DNAzymes. The affinity-related *K*_m_ value of minGAA is much higher (i.e. weaker affinity), resulting in a *k*_cat_*/K*_m_ of only ∼10^5^ M^–1^ min^–1^, which is far lower than those of the 10-23 DNAzyme (3.2 × 10^8^ M^–1^ min^–1^ even under low Mg^2+^ conditions [2]). For the 8-17 DNAzyme, *k*_cat_*/K*_m_ values of 1 × 10^5^–2 × 10^7^ M^–1^ min^–1^ were reported [30], and the lowest limit of this region is comparable with minGAA. Extending the arm regions could improve the apparent affinity (i.e. reduce *K*_m_), although this modification appeared to abolish multiple turnover activity (Fig. 2A).

For the pH dependence, the cleavage yield of minGAA increased as the pH rose from pH 6.0 to pH 7.5, but decreased sharply at pH 8.0 (Fig. 2B). We analyzed the enzymatic kinetics at these pH values (Supplementary Fig. S2) and found that the increase in the reaction rate accounted for the rise in cleavage yield up to pH 7.5. However, at pH 8.0, although the reaction rate was very high, the drastically reduced amplitude resulted in a low cleavage yield. This behavior is discussed in a later section in relation to the proposed catalytic mechanism.

We also examined the Zn^2+^ ion concentration dependency and found a maximum activity at 1 mM (Fig. 2C). This is in contrast to 10-23 DNAzyme where an increase of the Mg^2+^ concentration from 2 mM to 50 mM increased the* k*_cat_ value by ∼20-fold [2]. In addition, we examined the metal ion selectivity of minGAA by measuring the cleaved amount of the substrate in the presence of various metal ions. By testing the divalent metal ions Mg^2+^, Ca^2+^, Mn^2+^, Fe^2+^, Co^2+^, Cu^2+^, Zn^2+^, and Ba^2+^ and the monovalent ions Li^+^, Na^+^, and K^+^ as the cofactor, we found that minGAA DNAzyme is highly specific for Zn^2+^ (Fig. 2D). We examined whether the combination of Zn^2+^ and Mg^2+^ alters the enzymatic activity, as previously investigated for the IR3 DNAzyme [5], and we found that Mg^2+^ rather slightly reduced the reaction rate (0.049 min^–1^; Supplementary Fig. S3). This inhibitory effect may result from competition for binding at the catalytic site, as observed for leadzyme [31].

To determine where and how minGAA cleaves the substrate RNA, we performed LC-MS (Table 2, Supplementary Fig. S4). In this experiment, we used the *cis*-type minGAA molecule, in which the catalytic and substrate strands were combined via a hairpin-forming sequence (5′-GCGAAGC-3′) [32]. The molecular masses of the cleaved products indicated that minGAA cleaves the substrate at 5′-rG↓rArAG-3′, and also that the chemical structure at the 5′ end of the 3′ fragment is 5′-hydroxyl and the structure at the 3′ end is a mixture of 2′,3′-cyclic phosphate (5′-fragment 1) and 3′-phosphate (3′-PO_3_^−^, 5′-fragment 2).

**Table 2.** LC -MS analysis of the cleaved substrates by *cis*-type minGAA^a^

	Terminus	Mass (Da)	
		Theoretical	Observed
5′-Fragment 1	2′,3′-Cyclic phosphate	5612.7	5612.2
5′-Fragment 2	3′-Phosphate	5629.7	5629.8
3′-Fragment	5′-Hydroxyl	2450.7	2450.1

^a^Corresponding mass spectra are provided in Supplementary Fig. S4.

### Nucleotide requirements or preferences for minGAA and the substrate

**Figure 3.**
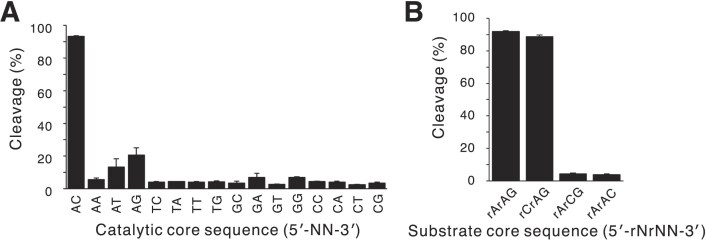
Sequence specificity in the catalytic core (**A**) and substrate core (**B**) for minGAA. The cleaved fractions of the substrate in the presence of 1 mM Zn^2+^ are shown. The error bars represent the SD of three independent experiments.

To elucidate the nucleotide preferences in the 2 nt catalytic core of minGAA, 15 mutants covering all the possibilities were produced and evaluated for their cleavage activities (Fig. 3A). Only the molecule with the 5′-AC-3′ sequence that was most enriched in the *in vitro* selection showed high activity, which clearly indicated that these two nucleotides are essential.

We also performed an *in vitro* selection experiment in order to elucidate the nucleotide preference for the catalytic core of minGAA and at the same time in order to obtain more active sequences (Supplementary Fig. S5). By using the N3-DNA library with randomization of the region corresponding to the 5′-ACC-3′ sequence of minGAA, we indeed observed that 5′-ACC-3′ was the most enriched, followed by 5′-ACA-3′. The observation is consistent with the above mutation experiment, and we confirmed that the sequence in the catalytic core of minGAA was the optimal one.

For the nucleotide specificity of the substrate of minGAA, we evaluated three different sequences with substitution of the respective bases in 5′-rArAG-3′ by cytosine (Fig. 3B). We did not use T or U as the replacement base because it can base-pair with the 5′-AC-3′ catalytic core. The result clearly indicated that the first rA, but not the following two, could be altered. Therefore, we concluded that the 5′-rAG-3′ sequence is essential as the substrate core sequence.

We also performed a library selection experiment to elucidate the substrate specificity (Supplementary Fig. S6), which showed that minGAA efficiently cleaved two substrate sequences, 5′-rArArG-3′ and 5′-rCrArG-3′, in the core substrate region and moderately cleaved the sequence 5′-UrArG-3′ (note that RNA is allowed for the last G, which is also true in the experiment in Supplementary Fig. S5). The result is consistent with the above point mutation analysis in that the 5′-rAG-3′ (or 5′-rArG-3′) sequence is strictly required. Also, it showed that the first rA can be substituted, although G at this position is not preferred, as it may form the G–C base pair with the catalytic core.

In addition, we examined an all-RNA substrate (sequence in Supplementary Table S1) and found that the rate was significantly reduced to ∼1/7 of that for the chimeric substrate, although the reaction still reached a high amplitude (Supplementary Fig. S7; Table 1). We also tested a single-RNA substrate, in which only the G nucleotide at the cleavage site was a ribonucleotide (Supplementary Table S1); this substrate showed very low activity (Supplementary Fig. S7; Table 1). Furthermore, using substrates with 5′-rArArAG-3′, 5′-rCrArAG-3′, or 5′-UrArAG-3′ sequences (Supplementary Table S1; Table 1; Supplementary Fig. S7), we confirmed that a G base at the cleavage site is essential for catalysis, consistent with the likely C8–rG18 base pairing.

Reasons for the above requirements or preference in the catalytic and substrate core sequences were explained via the 3D structures determined by X-ray crystallography and NMR spectroscopy, as detailed below.

### X-ray crystallography of minGAA

**Figure 4.**
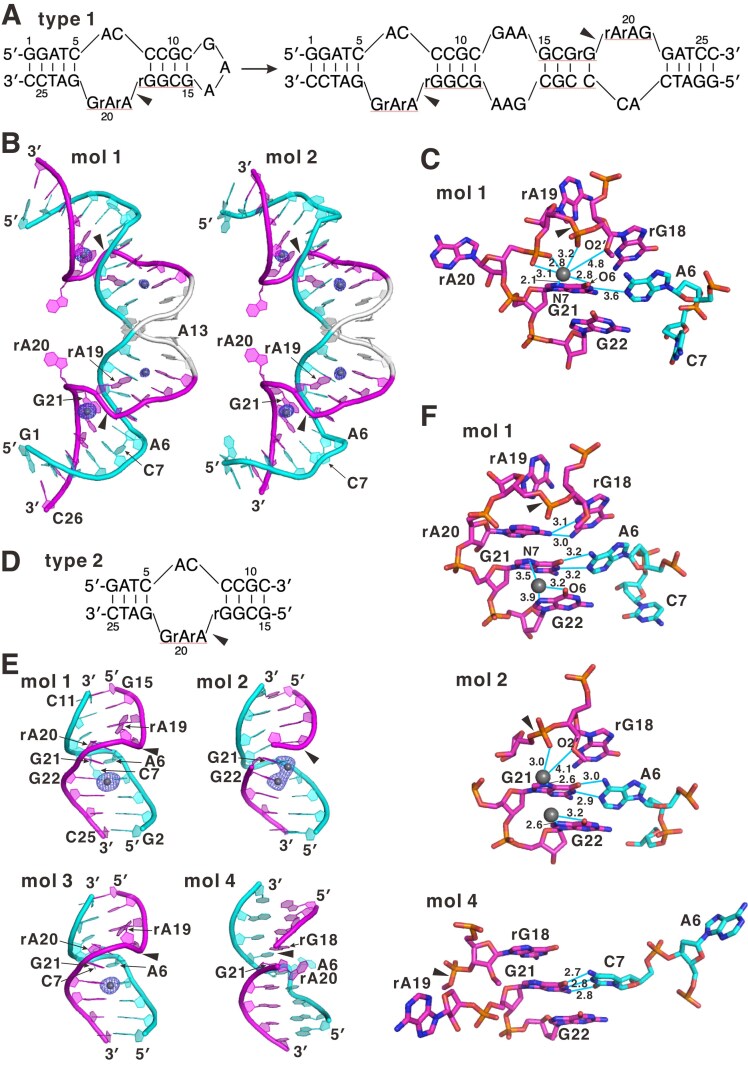
Crystal structures of two types of minGAA molecules. (**A**) Sequence of the type 1 construct, forming a hairpin (left) or double-strand configuration (right). (**B**) Structures of two molecules in an asymmetric unit of the crystal of the type 1 construct. (**C**) The major metal-binding site in molecule 1. (**D**) Sequence of the type 2 molecule. Nucleotides are numbered as in (A). (**E**) Structures of four molecules in an asymmetric unit of the crystal of the type 2 construct. (**F**) The metal-binding sites in molecules 1 and 2, and the corresponding site in molecule 4. The cleavage sites or bonds are shown by arrowheads, although O2′ of rG18 was methylated to deactivate the reaction. In (B) and (E), the regions corresponding to the catalytic and substrate strands are colored cyan and magenta, respectively. The metal ions are shown as spheres and are accompanied by the mFo–DFc omit maps contoured at 5.0 σ, except for molecule 2 in (E), which has half occupancies and is contoured at 3.5 σ. In (C) and (F), distances are indicated in Ångstroms. The molecular graphic figures were created by PyMOL ver. 1.8 or 2.5 (Schrodinger, LLC).

For crystallography, we used two molecular constructs of minGAA (Fig. 4). The type 1 construct was the same as that used in the LC-MS experiment except that 2′-*O*-methylation was introduced at rG18 to inactivate the catalysis. As with that for LC-MS, this molecule was intended to form a *cis*-type arrangement by introducing the 5′-GCGAAGC-3′ sequence which adopts a hairpin conformation in solution [32]. We determined the crystal structure of this molecule at 2.85 Å resolution (Supplementary Table S2), and unexpectedly found that it was in a dimeric form (Fig. 4A, B). The asymmetric unit of the crystal consisted of two of the dimeric molecules, which were very similar to each other. The structure of the region corresponding to a single unit of minGAA (a continuous span of the colored regions in Fig. 4B) showed the characteristics of B-DNA. Specifically, base pairs stacking nearly in parallel are observed continuously throughout the molecule, including the catalytic/substrate core in the center, which was initially thought to form a bulge. In this structure, A6 and G21 form a non-Watson–Crick G–A base pair with an *anti*–*anti* conformation [33] that intervenes between the C5–G22 and C8–G18 base pairs. However, the A6–G21 base pair is propeller-twisted and slightly too distant to be stabilized by hydrogen bonds (Fig. 4C). It is noteworthy that this twist in the base pair allowed A6 and G21 to stack closely to the C8–G18 and C5–G22 base pairs, respectively, ensuring the continuity of base pairing/stacking throughout the molecule. Consequently, one nucleotide of the catalytic strand (C7) and two nucleotides of the substrate strand (rA19 and rA20) are excluded from the continuous base pairing/stacking and protrude outside. Among them, rA19 and rA20 interact with the adjacent molecules in the crystal lattice by base stacking (Supplementary Fig. S8).

We observed two metal-binding sites in a single type 1 molecule (four in a dimer), with the major site having higher density located around the catalytic/substrate core (Fig. 4B, C). The metal at this site coordinated to the N7 atom of G21 with a distance of 2.1 Å (Zn–N coordination bond lengths are 2.1–2.5 Å, as seen in nucleic acid structures in the PDB, e.g. 1G6D, 1NLC, and 5DH8), and was stabilized by contacts with the negatively charged phosphate oxygens of rA19 and rA20. The distance to the O2′ atom of rG18 is 4.8 Å, which is too far to be considered as a direct contact, although a presumed water molecule in the hydration shell of Zn^2+^ might form a contact. Because the crystallization condition for the type 1 molecule included Sr^2+^ (see the Materials and methods), we cannot exclude the possibility that the identified metal-binding sites are for Sr^2+^ rather than Zn^2+^. However, a survey of the Protein Data Bank for interactions of Sr^2+^ with nucleic acids revealed that it rarely interacts with the N7 atom of G bases, as in Fig. 4C, and, when it does, the atomic distances are typically ≥2.5 Å. This suggests that the major site is more likely to bind Zn^2+^. Additionally, studies on leadzyme have shown that metals such as Ba^2+^ or Sr^2+^ can effectively mimic the catalysis involving the functional metal (Pb^2+^) [31, 34], indicating that at least this metal-binding site is valuable for understanding the catalytic mechanism in either case.

On the other hand, the crystallization condition for a shorter *trans*-type construct (type 2, Fig. 4D) included only Zn^2+^ as the heavy metal ion. Although light metals such as Mg^2+^ are also included, they can be easily distinguished from Zn^2+^ by the difference in electron density. The crystal structure of the type 2 molecule was determined at 3.04 Å resolution (Supplementary Table S2; Fig. 4E). The asymmetric unit contained four molecules. As in type 1, the overall structures showed characteristics of B-DNA, where the base pairs stacked in parallel, including A6–G21 (molecules 1–3) or C7–G21 (molecule 4), base-pair in the core catalytic/substrate site. In contrast to type 1, these base pairs are stabilized by hydrogen bonds (Fig. 4F), suggesting that hydrogen bonds should also form in type 1 if the molecule is released from crystal packing through base stacking at rA20. In molecules 1 and 3 of the type 2 construct, which are very similar to each other, rA20 is oriented inside, lying in the layer of the C8–rG18 base pair, forming hydrogen bonds with rG18, and stacking in parallel with G21. In contrast, C7 (or A6 for molecule 4) and rA19 are excluded from the base pairing/stacking and protrude outside or are invisible due to flexibility. As in type 1, the protruding bases, i.e. rA19 (molecules 1, 3, and 4) and A6 (molecule 4), underwent base stacking with the adjacent molecules of the crystal lattice (Supplementary Fig. S8). A metal-binding site was identified between the G21 and G22 bases in molecules 1 and 3 (Fig. 4E, F), while two alternative sites, each with 50% occupancy, were identified at G21 and G22 bases in molecule 2. Among these sites, that at G21 corresponds to the major site of the type 1 molecule. In contrast, we detected no metal-binding site in molecule 4.

The above observations for the molecules of the type 1 and type 2 constructs are largely consistent with each other, i.e. in terms of the B-DNA-like overall structure, the G–A base pairing in the core region, and metal binding at G21. However, details are substantially inconsistent especially for the conformation of rA19 and rA20, which should be ascribed to the intrinsic flexibility of this region and crystal packing forces. Therefore, we determined the structure in solution by NMR spectroscopy, and compared it with the crystal structures, before discussing the roles of the nucleotides and catalytic mechanism.

### NMR solution structure of minGAA

**Figure 5.**
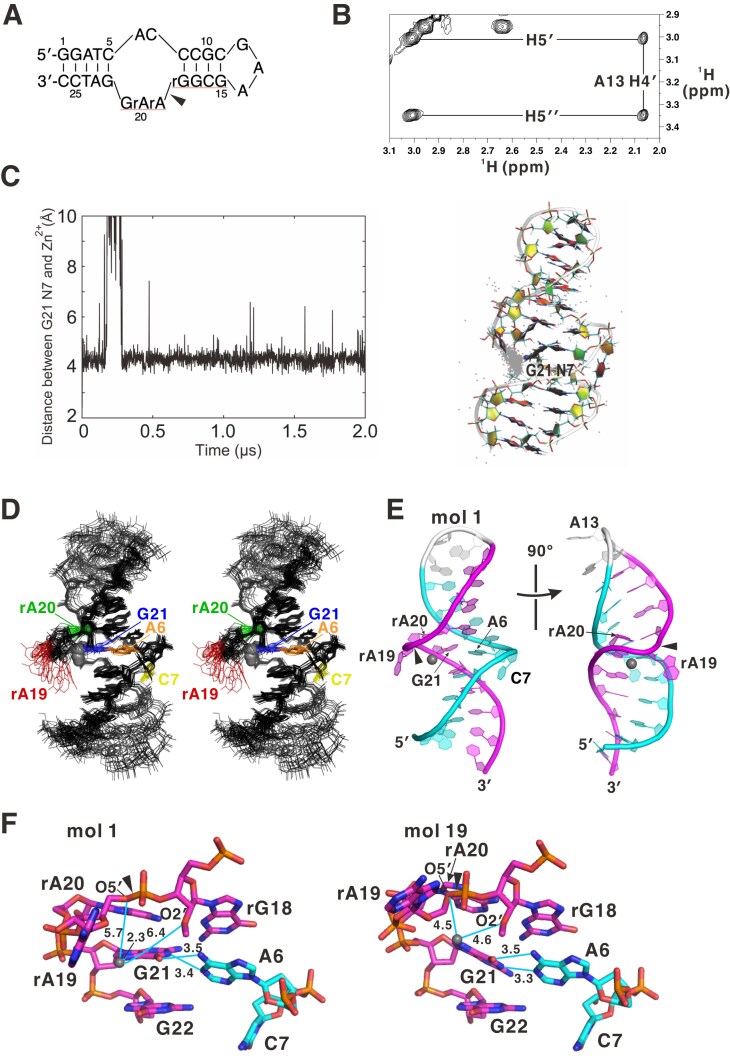
NMR solution structure of minGAA. (**A**) Sequence of the type 1 molecule used in the NMR analysis. (**B**) A part of the NOESY spectrum showing the cross-peaks of H4′ and H5′ atoms of A13. (**C**) Distance between Zn^2+^ and G21 N7 (left panel) and distribution of a Zn^2+^ ion (gray dots in the right panel) during the MD simulation of the behavior of Zn^2+^ toward a preliminary Zn^2+^-free NMR structure. The figure was created by VMD [36]. (**D**) The ensemble of the 20 selected structures in a stereo view. The central part (T4–C9/G17–A23) except for flexible rA19 was aligned. (**E**) The structure of molecule 1 with minimal energy is shown where the orientation in the left panel is the same as that in (D). (**F**) The Zn^2+^-binding site in molecules 1 and 19. In (A), (E), and (F), the cleavage sites or bonds are shown by arrowheads. The molecular graphics in (D–F) were created by PyMOL ver. 2.5.

For analysis by NMR, we used the type 1 construct (Fig. 5A) that was also used in crystallography. In solution, the molecule was likely to adopt a hairpin conformation as indicated by the extremely upfield-shifted peak (2.06 ppm) of H4′ of A13 (Fig. 5B), similar to the isolated DNA of the 5′-GCGAAGC-3′ sequence (2.19 ppm for the equivalent proton; 4.17–4.50 ppm for the H4′ atoms of the other nucleotides) [32]. This shift was caused by the ring-current effect [35], where this proton is located on the purine ring of A14. It only occurs in the hairpin conformation, but not in the double strand as in the crystal structure.

The NMR resonances were assigned based on the nuclear Overhauser effects (NOEs) between adjacent nucleotides [35], inter-strand NOEs, ^1^H–^13^C coherences, and RNA/DNA mutations at rA19 and rA20 (Supplementary Fig. S9). We collected structure restraints (Supplementary Table S3), and calculated a preliminary structure without a Zn^2+^ ion. In order to estimate the Zn^2+^-binding site in solution, we performed MD simulations, which can reveal the dynamics of biomolecules and sometimes provide novel insights into unknown conformations. The results indicated that the Zn^2+^ ion is most stably positioned near the N7 atom of G21 (Fig. 5C; see the Supplementary Movies), even though the possibility of a coordination bond was not considered in the simulation, suggesting that the position probably stabilized the cation via electrostatic attraction. This site is essentially the same as the major metal-binding site in the crystal structure of the type 1 construct and one of the two alternative sites in molecule 2 of the type 2 construct (Fig. 4C, F). Therefore, we calculated the structure of minGAA containing a Zn^2+^ ion bound at this site, coordinating with the G21 N7 atom (Fig. 5D, E; Supplementary Table S3). As with the crystal structures, the determined solution structure has characteristics of B-DNA, with a slight bend in the middle, where the A6–G21 base pair mediated the continuous base stacking throughout the molecule.

It is remarkable that the position of the rA19 base varied widely among the structural ensembles (Fig. 5D). This indicates intrinsic flexibility of the nucleotide, which is fully consistent with the observation in the crystal structures. Namely, positions of rA19 in crystals varied by forming base–base stacking with adjacent molecules in the crystal lattice or were otherwise invisible. In contrast, rA20 was stably located within the groove, typically stacking with G21 (Fig. 5D–F). This position is equivalent to molecule 1 (and molecule 3) in the type 2 crystal structure (Fig. 4F). Additionally, the position of C7 is largely fixed within the groove, even though it is excluded from the base pairing, which is similar to molecules 1 and 3 of the type 2 crystal structure.

The Zn^2+^ ion, coordinating with the G21 N7 atom, is surrounded by the flexible and negatively charged sugar-phosphate backbone of rA19–rA20 (Fig. 5F). This arrangement is similar to that observed in the type 1 crystal structure (Fig. 4C) and at one of the two sites of molecule 2 in the type 2 crystal structure (Fig. 4F). This is likely to explain the determinants of Zn^2+^ location at this site via electrostatic attraction. Noticeably, in the structural ensembles, the distances from Zn^2+^ to rG18 O2′ and rA19 O5′ at the cleavage site are 4.6–7.1 Å and 4.3–5.7 Å, respectively. The Zn^2+^ ion coordinated to the N7 atom of a guanine base typically exhibits an octahedral hydration shell with a coordination number of six, with one site occupied by N7 (as observed in PDB entries 1G6D and 1NLC). Considering the Zn^2+^–O bond length of ∼2.1 Å for this geometry [37], these distances probably allow occasional hydrogen-bonding contacts involving the water molecules in the shell. In particular, hydrogen bonds with O–O distances of ≤2.5 Å, as allowed in this case, are considered low-barrier hydrogen bonds that can facilitate proton (and electron) transfer [38, 39].

Although the solution structure does not perfectly coincide with any of the crystal structures, they share major common features: (i) B-DNA-like continuous stacking of base pairs, including A6–G21; and (ii) interaction of Zn^2+^ coordinated at G21 N7 with the backbone around the cleavage site. Consequently, the solution structure can be viewed as the result of removing crystal packing forces present in the crystal structures. For example, in molecule 1 of the type 2 crystal structure (Fig. 4F), rA19 (but not rA20) is involved in crystal packing. If rA19 were released from the packing forces, it would become flexible, allowing its sugar-phosphate backbone to form stabilizing contacts with Zn^2+^ coordinated at the G21 N7 atom.

### Structural bases for nucleotide requirements

Based on the 3D structures, the requirements for the nucleotides of the catalytic and substrate core (Fig 3), i.e. the roles of these required nucleotides, can be understood as follows. Adenosine at position 6 of the catalytic core is strongly required because it is involved in the A6–G21 non-Watson–Crick base pair, which is essential for stabilizing the B-DNA-like structure. Cytidine at position 7 is also required, which may be related to the good fitting of the C7 base in the groove as observed in the solution structure (Fig. 5D, E) and some molecules in the crystal structures (the type 1 construct, and molecules 1 and 3 of the type 2 construct; Fig. 4B, E). The role of C7 is probably to be stably positioned after being excluded from the base pair/stacking group, ensuring the stacking of A6–G21 and C8–rG18 base pairs. The reduction of the reaction rate for all-RNA substrate (Table 1; Supplementary Fig. S7) can be explained by the importance of a B-DNA-like structure, in which the DNA/RNA hybrid in the arm regions probably adopts an intermediate conformation between A- and B-forms [40]. The requirement for rG18 (Table 1; Supplementary Fig. S7) is explained by the formation of the C8–rG18 base pair.

For the substrate core, only rA19 can be replaced by a nucleotide other than rG (Fig. 3B; Supplementary Fig. S3), which is explained well by the flexibility of this base without interaction with other bases. Guanine may form a base pair with C7, disrupting the geometry of the Zn^2+^-binding site. In contrast, rA20 is required because its stacking with G21 stabilizes the Zn^2+^-binding geometry, and presumably the hydration shell. The requirement for G21 as the major Zn^2+^-binding site is obvious. The experiment with the single-RNA substrate (Table 1; Supplementary Fig. S7) suggested that either or both of these two adenosines must be ribonucleotides, which can be rationalized by the fact that their O2′ atoms point outwards, probably allowing preferable interactions with water molecules.

### Catalytic mechanism

**Figure 6.**
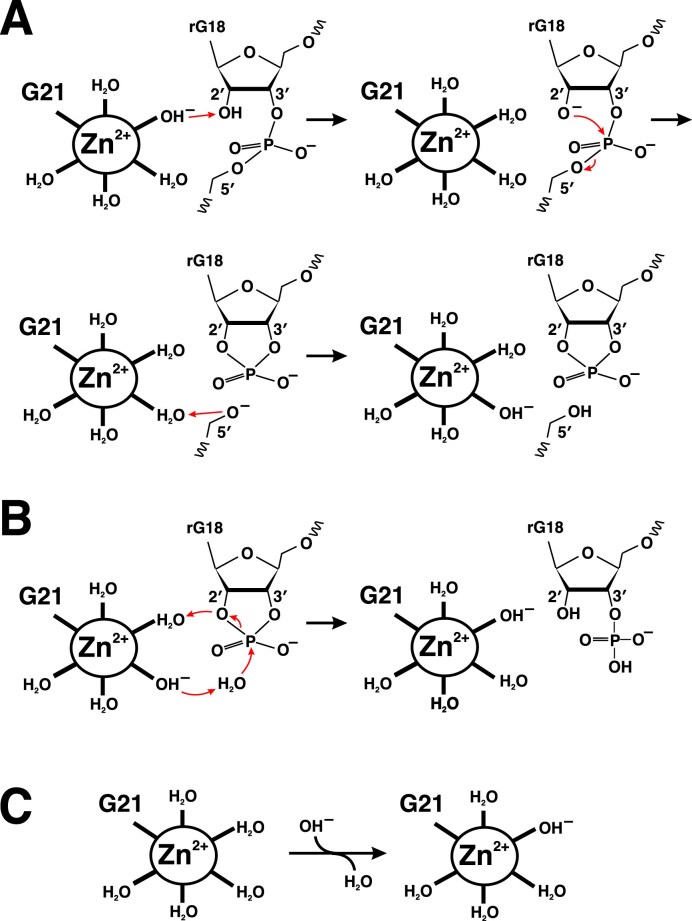
Proposed catalytic mechanism of minGAA. (**A**) The main reaction that generates 2′,3′-cyclic phosphate. (**B**) The second reaction that hydrolyzes the 2′,3′-cyclic phosphate. The red arrows show the routes of redistribution of a negative charge. (**C**) Probable conversion of the hydration shell of the initially bound Zn^2+^ ion.

As the zinc ion species, Zn^2+^ and Zn(OH)^+^ (one OH^–^ hydrated to Zn^2+^) co-exist in aqueous solution at the optimal pH of minGAA, i.e. pH 7.0–7.5 (Fig. 2B) [41]. As described earlier, the number of water molecules fully coordinated to Zn^2+^ is six [37], which is typical for coordination to guanine N7. The Zn^2+^ and Zn(OH)^+^ ions are expected to exist as Zn^2+^(H_2_O)_6_ and Zn^2+^(H_2_O)_5_(OH^–^), respectively, in which one of the water molecules is replaced by G21 N7 in the minGAA molecule (Fig. 6). As mentioned above, in both the crystal and solution structures, the water molecules (or a hydroxide ion) in the hydration shell are capable of forming low-barrier hydrogen bonds (O–O distance of ≤2.5 Å) [38, 39] with the rG18 O2′ and rA19 O5′ atoms at the cleavage site, at least occasionally.

These conditions led us to propose a Zn^2+^-dependent catalytic mechanism, based on the general acid–base scheme adopted for hammerhead and hairpin ribozymes (Fig. 6A) [42–44]. In this mechanism, the hydroxide ion in Zn(OH)^+^ acts as a base, drawing a proton from (and conferring a negative charge to) the 2′-OH of rG18, thereby generating a negatively charged O^–^. The resultant O^–^ then attacks the phosphorus center, breaking the P–O5′ bond and leaving a negative charge on the O5′ atom. Subsequently, a water molecule coordinated to Zn^2+^ acts as an acid, donating a proton to O5′ and neutralizing its charge. In the final product, the strand is cleaved to yield 2′,3′-cyclic phosphate and 5′-hydroxyl termini, as observed in the LC-MS experiment (Table 2; Supplementary Fig. S4). Along with this cascade of the charge redistribution, the negative charge of OH^–^ in Zn(OH)^+^ is transiently transferred to the nucleic acid strand and then returns, causing the zinc ion bound to minGAA to oscillate between Zn(OH)^+^ and Zn^2+^.

There should be a subsequent reaction that hydrolyzes the 2′,3′-cyclic phosphate to generate 3′-phosphate (Fig. 6B), which occurs partially, as indicated by the MS results (Table 2). We propose that the hydroxide ion of Zn(OH)^+^ is involved in the nucleophilic attack on phosphorus by a water molecule.

It should be noted that the stabilities (or proportions) of the two zinc ion species are critical factors in these reactions. Specifically, the strength required to abstract a proton in the first step depends on the stability of Zn^2+^ without a hydroxide ion, whereas the ability to donate a proton in the final step relies on the stability of Zn(OH)^+^. We propose that this balance between the two species determines the active pH range of minGAA.

We should point out, however, another aspect: Zn^2+^ may bind more stably to the DNAzyme than Zn(OH)^+^, because the negatively charged phosphate groups at the binding site stabilize the more positively charged species. It is possible that conversion of the hydration shell of Zn^2+^ after its binding to the DNAzyme (Fig. 6C) is required prior to the catalytic cascade shown in Fig. 6A. If this process represents the rate-limiting step, the kinetic behaviors observed at different pH values (Supplementary Fig. S2) can be explained. Specifically, the increase in the reaction rate from pH 6 to pH 7.5 can be attributed to the rise in the concentration of OH^–^. At pH 8.0, although the rate increases further, the fraction of Zn^2+^ bound to the DNAzyme decreases, reflecting the solution composition, and consequently the cleavage yield becomes low.

### Comparison with other structures

Currently the 3D structures of three DNAzymes (9DB1, 8-17, and 10-23) are available (Supplementary Fig. S10A–C) [16–18, 20]. These DNAzymes have catalytic core or loop regions consisting of 15–31 nucleotides, while their corresponding substrate regions are only 1–2 nucleotides. Due to this large discrepancy, their overall structures kink in the middle, causing the two double-stranded flanking regions to orient at approximately right angles to each other. This contrasts with the B-DNA-like overall structure of minGAA (Figs 4 and 5), probably because the catalytic and substrate core regions of minGAA differ by only one nucleotide.

In this regard, minGAA is similar to a Pb^2+^-dependent ribozyme, leadzyme, whose structure has been determined both in crystal form and in solution (Supplementary Fig. S10D, E) [31, 34, 45]. Probably because the catalytic and substrate core regions differ by only two nucleotides (2 and 4 nt, respectively), the overall structure of leadzyme is also B-DNA-like. Although the base pairing of the core region was disrupted by the flipping out of substrate nucleotides, base stacking in the catalytic strand appeared to be largely maintained. For minGAA, with the G–A base pair in the core region, both base stacking and base pairing are observed throughout the structure, as described earlier (Figs 4 and 5).

In the catalytic site of leadzyme, Sr^2+^, which mimics Pb^2+^, is stabilized by interactions with the base and phosphate atoms of both the catalytic and substrate strands (Supplementary Fig. S10F). The metal ion showed a close contact with O2′ of the cleavage site, where a hydroxide ion coordinated to Pb^2+^ may retrieve H^+^ from O2′, as proposed in the literature [34], similarly to the first half of the possible catalytic mechanism of minGAA described above. However, the distance between Pb^2+^ and O5′ is too long in the leadzyme structure (Supplementary Fig. S10F), in contrast to minGAA. Therefore, the negative charge at O5′ resulting from the leadzyme reaction is likely to be neutralized by a free water molecule or a hydronium ion.

## Conclusion

In the present study, we developed an RNA-cleaving DNAzyme, minGAA, which possesses a remarkably compact catalytic core composed of only two nucleotides and which efficiently cleaves a substrate core of just three nucleotides. Leadzyme is also considered a minimal ribozyme, containing catalytic and substrate core sequences of two and four nucleotides, respectively [46]. In addition, a recently discovered RNA-ligating ribozyme has three nucleotides each for its catalytic and substrate core regions [47]. Another RNA ligase ribozyme appears to possess only a two nucleotide catalytic core sequence, although it is likely to be assisted by separate loop regions [48]. For DNAzymes, the shortest known catalytic core is 10 nucleotides for I-R3 [5]. Therefore, minGAA is the smallest DNAzyme identified to date and, remarkably, is also among the smallest nucleic acid enzymes overall. The discovery of such a small DNAzyme, together with the structural elucidation of its catalytic mechanism, provides new insights into nucleic acid catalysis and may promote further advances in this field.

Because minGAA cleaves an all-RNA substrate slowly but nearly completely, it may be useful for the digestion of mRNA molecules. However, as Zn^2+^ is toxic, its therapeutic use inside cells is challenging. In contrast, minGAA may be useful *in vitro*, for example to assess mRNA quality in vaccine development.

## Supplementary Material

gkaf1502_Supplemental_Files

## Data Availability

The coordinates and structure factor data were deposited in the PDB under accession IDs 9k8p (type 1) and 9k8o (type 2). Coordinates and chemical shift values were deposited in the PDB and BMRB under accession IDs 9k8n and 36699. Other data underlyng this article are available in the article and in its online supplementary data.
