# Supplementary material for: A minimal RNA-cleaving DNAzyme and its catalytic mechanism
Source: Nucleic Acids Res. 2026 Jan 15;54(2):gkaf1502. doi: 10.1093/nar/gkaf1502 (PMC12805900; doi:10.1093/nar/gkaf1502)
Supplement: gkaf1502_Supplemental_Files [file gkaf1502_supplemental_files.zip › Supplementary_251111.docx]

**Supporting Information**

**A minimal RNA-cleaving DNAzyme and its catalytic mechanism**

Kazuhiko Yamasaki, Rika Inomata, Tomoko Yamasaki, Tomomi Kubota,

Naoyuki Miyashita, Koh Takeuchi, and Makoto Miyagishi

**Contents of Supporting Information**

Supplementary Methods

Kinetic experiments

Crystallography

NMR structure calculation

Supplementary Table

Table S1: Sequences of nucleic acids used in this study.

Table S2: Data collection and refinement statistics in crystallography.

Table S3: Structural statistics for NMR structure determination.

Supplementary Figure

Figure S1: Kinetic experiments for intermediate molecules during minimization of DNAzyme.

Figure S2: Kinetic experiments for minGAA (minGAA-s) at different pH.

Figure S3: Kinetic experiments for minGAA (minGAA-s) in the presence of Zn^2+^ and Mg^2+^ ions.

Figure S4: LC-MS of the fragments of the *cis*-type hairpin minGAA after cleavage.

Figure S5: Optimization of minGAA catalytic sequence by *in vitro* selection.

Figure S6: Preferred substrate core sequence of minGAA analyzed by using a random-nucleotide library.

Figure S7: Kinetic experiments for minGAA (minGAA-s) with modified substrates.

Figure S8: Base stacking observed between adjacent molecules in the crystal lattice.

Figure S9: NMR spectra for resonance assignments.

Figure S10: Crystal and solution structures of related DNAzymes and ribozymes.

Figure S11: The distribution of Na⁺ ions during the 2-μs MD simulation of the preliminary Zn²⁺-free NMR structure of minGAA.

Supplementary Movies

Movie S1: two micro-second MD simulation of preliminary Zn^2+^-free minGAA.

Movie S2: two micro-second MD simulation of preliminary Zn^2+^-free minGAA in the presence of Zn^2+^.

Supplementary Methods

*Kinetic experiments.*

The RNA-cleavage reaction was carried out under a single turnover condition with a 10-fold excess of the DNAzyme (1 μM) to the fluorescein (FAM)-labeled substrate (0.1 μM). The FAM-labeled substrate and DNAzymes were heated at 95°C for 5 min and allowed to cool at 4°C. The cofactor (zinc ion) was added to initiate the reaction. The DNAzymes annealed with the substrate were incubated at 37°C for a designated period time (typically, 0 min, 3 min, 10 min, 30 min, and 60 min) in the reaction buffer: 50 mM HEPES (pH 7.5), 150 mM NaCl and 1 mM ZnCl_2_. The reaction was terminated by an addition of a stop solution (25 mM EDTA, 8 M urea and 0.025% bromophenol blue). The cleaved products were separated by electrophoresis on a denaturing gel (20% polyacrylamide/bis and 7 M urea). The cleaved fractions were quantified using a ChemiDoc^TM^ XRS+ imaging system (Bio-Rad, Hercules, CA, USA) with 535–585 filter from the gel images.

The data was fit by using an in-house python3 program to an exponential equation

$f_{\left( t \right)}=f_{max}\left( 1-e^{{(-k}_{obs}\cdot t)} \right)$ (1)

where *f* is the cleaved fraction, *f_max_* is the amplitude (%), *t* is the time, and *k_obs_* is the reaction rate constant (min^−1^). The average and the standard deviation were obtained by a 1000-times bootstrap method employing randomly selected fraction data for a time from triplicate experiments. For a low reactive enzyme, a fixed value for amplitude (85%, considering results for similar enzymes/substrates) was used during calculations.

To obtain *k_cat_* and *K*_m_ values, the experiments were conducted under multiple turnover conditions (DNAzyme: substrate=1: 5, 10, 20, 40 and 80) in the above reaction buffer at 37°C. The *V*_0_ values were obtained by equation

*V*_0_ = *k_obs_* [S] $f_{max}$/100 (2)

where *k_obs_* and *f_max_* were obtained by exponential fitting of the cleaved fraction to **Eq. (1)**, and [S] is the initial concentration of substrate. The average and the standard deviation for *V*_0_ were also obtained by a 1000-times bootstrap fitting of the triplicate experiments. The relation between *V*_0_ and [S] was fitted to the Michaelis-Menten equation

*V*_0_ = *k_cat_* [E] [S] /(*K*_m_+[S]) (3)

where [E] is the concentration of enzyme. The average and the standard deviation for *k_cat_* and *K*_m_ were also obtained by a 1000-times bootstrap fitting using standard deviations for *V*_0 ,_ by using an in-house Python3 program.

*Crystallography.*

Type-1 minGAA molecule for structure determination (*cis*-minGAA-type-1 in Table S1) was crystallized in the mixture of 0.8 μL each of 1 mM nucleic acid dissolved in 5 mM 4-(2-hydroxyethyl)piperazine-1-ethanesulfonic acid (HEPES) (7.0) solution containing 1 mM ZnCl_2_ and a crystallization solution [6.75% (+/–)-2-methyl-2,4-pentandiol (MPD; Hampton, Aliso Viejo, CA, USA), 50 mM guanidine hydrochloride, 18 mM HEPES (7.5), 5.4 mM spermine, 18 mM LiCl, 36 mM SrCl_2_, and 4.5 mM MgCl_2_] by hanging-drop vapor diffusion against a solution containing 31.5% MPD and 100 mM guanidine hydrochloride. After incubation at 20 °C for two months, plate-like crystals of the widths of ~0.1 mm and thickness of ~0.03 mm were formed.

Type-2 minGAA molecule (minGAA-s-type-2/sub-type-2 in Table S1) was crystallized in the mixture of 0.15 μL each of 1 mM nucleic acid dissolved in 5 mM HEPES (7.0) solution containing 1 mM ZnCl_2_ and a crystallization solution [35% MPD, 40 mM sodium cacodylate (7.0), 12 mM spermine, 80 mM NaCl, 12 mM KCl, and 20 mM MgCl_2_] in the Natrix-2 kit (Hampton) by hanging-drop vapor diffusion against the above crystallization solution. After incubation at 20 °C for 2 days, rod-like crystals of the lengths of 1.5 mm and widths of 0.3–0.5 mm were formed.

Diffraction data were collected at the BL-1A beamline in the Photon Factory, KEK (Tsukuba, Japan). Crystal parameters were obtained by processing the data using XDS^1^ (Table S2). The diffraction phase was determined by molecular replacement using the Phaser module^2^ of the CCP4 program package.^3^ For type-2 molecule, the corresponding part of the NMR structure calculated without Zn^2+^ was used as the template model. For type-1 molecule, structure of type-2 molecule was used as the template. For refinement and model building, Refmac5^4^ and Coot^5^ were used.

*NMR analyses.*

Solution consisted of 0.4–0.5 mM nucleic acid, 10 mM d_11_-Tris-HCl (pH 7.0) (Isotec Inc.), 1 mM ZnCl_2_, 0.1 mM sodium 2,2-dimethyl-2-silapentane-5-sulfonate (DSS, Sigma), and 5% D_2_O.

A series of homonuclear and heteronuclear spectra,^6^ i.e., NOE spectroscopy (NOESY), total correlation spectroscopy (TOCSY), double-quantum-filtered correlation spectroscopy (DQF-COSY), and ^1^H–^13^C heteronuclear single-quantum correlation spectroscopy (HSQC) were recorded on Bruker Avance III-900 (900.05 MHz for ^1^H, 226.31 MHz for ^13^C) or Avance III-800 (800.13 MHz for ^1^H) spectrometers at 298 K, where mixing times of 100 ms and 50 ms for NOESY and TOCSY, respectively. Chemical shifts were directly or indirectly referenced to the internal DSS.^7^

The processed spectra were analyzed by Felix ver. 2007 (Felix NMR, Inc., San Diego, CA). The backbone and side chain ^1^H resonances were assigned on the basis of the typical base-sugar sequential connectivities.^6^ For the substrate loop of rA19–rA20, two mutants to DNA counterparts, respectively, are used to confirm the assignments.

A stepwise structural calculation protocol and restraints used are based on our previous NMR structure determination of the complex of a Zn^2+^-binding protein and a DNA duplex.^8^ The distance constraints derived from the NOESY spectra were classified into four categories, 1.5–2.8, 1.5–3.5, 2.0–4.5, and 2.5–6.0 Å, according to the relationship that NOE intensity is inversely proportional to the sixth power of distance. The average intensities of the intra-nucleotide NOEs of H5–H6 pairs of C bases were used for the calibration of the intensities.

For DNA backbone, theoretical restraints were applied to the B-DNA regions (G2–C5, C8–C11, G15–G17, and G22–C25). Specifically, α, β, and ζ torsion angles were weakly restrained to –60°±30°, 180°±30°, and –90°±30°, respectively, under the assumption of B-form DNA conformation^9^. In addition, ε torsion angles were restrained to stereochemically allowed range, 225°±75°^10^. To maintain hydrogen bonds in the typical Watson-Crick base pairs, N1–N3/H3 and N6/H61–O4 atoms of A–T base pairs and N1/H1–N3, N2/H21–O2, and O6–N4/H41 atoms of G–­C base pairs were restrained. The donor-acceptor and proton-acceptor distances were set to 2.5–3.0 Å and 1.5–2.0 Å, respectively. Also, planarity restraints were applied for all nonhydrogen atoms included in the same Watson-Crick base pairs. In addition, hydrogen bonding in the non-Watson-Crick base pairs of A6–G21 (N1–N1/H1 and N6/H61–O6) at the catalytic/substrate core region and G12–A14 (N2/H22–N7 and N3–N6/H62) at the hairpin edge were restrained by donor-acceptor and proton-acceptor distances of 2.5–3.5 Å and 1.5–2.5 Å, respectively. These theoretical restraints were carefully introduced after observing that the structures calculated without the restraints adopted such conformations.

To maintain the zinc coordination by the G21 base, a restraint was imposed on the Zn^2+^–N7 distance by 2.1–2.3 Å. Also, a planar restraint was introduced to Zn^2+^ and nonhydrogen atoms of the G21 base.

Force constants were 75–150 kcal mol^–1^ Å^–2^ for the distance constraints, 5–400 kcal mol^–1^ rad^–2^ for the dihedral angle restraints, and 1.0 kcal mol^–1^ Å^–2^ for the planarity restraints.

Structure calculations were carried out by using CNS^11^ ver. 1.3 in two stages. In the first stage, a random simulated annealing^12^ by the torsion angle molecular dynamics was performed in the absence of Zn^2+^, where the NOE distance restraints and the theoretical dihedral, distance, and planarity restraints were applied. A high temperature simulated annealing at a temperature of 20,000 K for 10,000 steps, and a slow-cool simulated annealing at 20,000 K to 0 K for 10,000 steps were conducted. In addition, Cartesian molecular dynamics of a slow-cool annealing at 2,000 K to 0 K for 3,000 steps was performed, which was followed by an energy minimization.

In the second stage, simulated annealing by the Cartesian molecular dynamics was performed for the Zn^2+^-bound DNAzyme. The restraints were the same as in the first stage, except that the theoretical constraints regarding the Zn^2+^-coordination were included. A constant temperature annealing at 4,000 K for 10,000 steps and a slow-cool annealing at initially 4,000 K for 10,000 steps were performed, which was followed by a final energy minimization.

From 100 initial structures, 20 structures with no distance violation larger than 0.2 Å, no torsion angle violation larger than 2 degrees, no inter atomic distance less than 1.4 Å, and the lowest total energies were selected as the final accepted structures. The minimized mean structure was produced by a protocol for the selection of the accepted structures in the CNS program.

The average RMSD of the co-ordinates from the unminimized mean structure of the ensemble were calculated using the program PyMOL and an in-house Fortran 77 program. The experimental restraints and stereochemical properties of the NMR solution structure are shown in Table S3.

**References**

(1) Kabsch, W. *Acta Crystallogr. D Biol. Crystallogr.* **2010**, *66*, 125-132.

(2) Mccoy, A. J.; Grosse-Kunstleve, R. W.; Adams, P. D.; Winn, M. D.; Storoni, L. C.; Read, R. J. *J Appl Crystallogr* **2007**, *40*, 658-674.

(3) Potterton, E.; Briggs, P.; Turkenburg, M.; Dodson, E. *Acta Crystallographica Section D-Biological Crystallography* **2003**, *59*, 1131-1137.

(4) Murshudov, G. N.; Vagin, A. A.; Dodson, E. J. *Acta Crystallographica Section D-Biological Crystallography* **1997**, *53*, 240-255.

(5) Emsley, P.; Lohkamp, B.; Scott, W. G.; Cowtan, K. *Acta Crystallographica Section D-Biological Crystallography* **2010**, *66*, 486-501.

(6) Wüthrich, K. *NMR of Proteins and Nucleic Acids*; John Wiley & Sons, Inc.: New York, 1986.

(7) Wishart, D. S.; Bigam, C. G.; Yao, J.; Abildgaard, F.; Dyson, H. J.; Oldfield, E.; Markley, J. L.; Sykes, B. D. *J. Biomol. NMR* **1995**, *6*, 135-140.

(8) Yamasaki, K.; Kigawa, T.; Watanabe, S.; Inoue, M.; Yamasaki, T.; Seki, M.; Shinozaki, K.; Yokoyama, S. *J. Biol. Chem.* **2012**, *287*, 7683-7691.

(9) Tjandra, N.; Tate, S.-i.; Ono, A.; Kainosho, M.; Bax, A. *J. Am. Chem. Soc.* **2000**, *122*, 6190-6200.

(10) Phan, A. T.; Kuryavyi, V.; Ma, J.-B.; Faure, A.; Andréola, M.-L.; Patel, D. J. *Proceedings of the National Academy of Sciences* **2005**, *102*, 634-639.

(11) Brünger, A. T.; Adams, P. D.; Clore, G. M.; DeLano, W. L.; Gros, P.; Grosse-Kunstleve, R. W.; Jiang, J. S.; Kuszewski, J.; Nilges, M.; Pannu, N. S. *et al.* *Acta Crystallographica Section D-Biological Crystallography* **1998**, *54*, 905-921.

(12) Nilges, M.; Clore, G. M.; Gronenborn, A. M. *FEBS Lett.* **1988**, *239*, 129-136.

(13) Humphrey, W.; Dalke, A.; Schulten, K. *J. Mol. Graph. Model.* **1996**, *14*, 33-38.

**Table S1: Sequences of nucleic acids used in this study.**

| **Libraries used for *in vitro* selection experiments (5´ to 3´)** | |
| --- | --- |
| N10-DNA | CCA TAC ATC GTT TGC ATC NNN NNN NNN NGT CTG CCT AGT TCA GAT |
| N3-DNA | ACG TTT GCT ACA TAC CTC NNN GTA GAC TTG ATC CGT CT |
| rN3-Sub | TTG CTA CAT ACC ACG CAG ACG TT rGrNrNrN GAT GCA CTT TGG AGA ACT TGA TCC |
|  |  |
| **Primers used for amplification (5´ to 3´)** | |
| N10-DNA-F | CGT CTC TCC GAA CCA TAC ATC GTT TGC AT |
| N10-DNA-R | CAC CTT TCC GAA ATC TGA ACT AGG CAG AC |
| N3-DNA-F | GTG GAG AGG TTC TTA CAA CGT TTG CTA CAT ACC TC |
| N3-DNA-R | GCG GAG AGG CTC TCA CAA GAC GGA TCA AGT CTAC |
| rN3-Sub-F | GTG GAG AGG TTC TTA CAT TGC TAC ATA CCAC |
| rN3-Sub-R | GCG GAG AGG CTC TCA CAG GAT CAA GTT CTCC |
|  |  |
| **DNAzymes used in this study (5´ to 3´)** | |
| IR3-R | AAG TGC ATC ACC ACG CAG AGT CTG CCT AGT TCA GA |
| IR3-R-wo4 | AAG TGC ATC ACC ACG GTC TGC CTA GTT CAG AT |
| minGAA | AAG TGC ATC ACC AAC GTC TGC CTA GTT CAG AT |
| minGAA-iACGT | AAG TGC ATC ACC AAC ACG TGT CTG CCT AGT TCA GAT |
| minGAA-iGATC | AAG TGC ATC ACC AAC GAT CGT CTG CCT AGT TCA GAT |
| IR3 | AAG TGC ATC TAG TTG AGC TGT CTG CCT AGT TCA GAT |
| *cis*-minGAA | GGA TCA CCC GCG AAG CGrG rArAG GAT CC |
| minGAA-s | AAG TGC ATC ACC AAC GTC TG |
| minGAA-AA | AAG TGC ATC AAC AAC GTC TG |
| minGAA-AT | AAG TGC ATC ATC AAC GTC TG |
| minGAA-AG | AAG TGC ATC AGC AAC GTC TG |
| minGAA-TC | AAG TGC ATC TCC AAC GTC TG |
| minGAA-TA | AAG TGC ATC ATC AAC GTC TG |
| minGAA-TT | AAG TGC ATC TTC AAC GTC TG |
| minGAA-TG | AAG TGC ATC TGC AAC GTC TG |
| minGAA-GC | AAG TGC ATC GCC AAC GTC TG |
| minGAA-GA | AAG TGC ATC GAC AAC GTC TG |
| minGAA-GT | AAG TGC ATC GTC AAC GTC TG |
| minGAA-GG | AAG TGC ATC GGC AAC GTC TG |
| minGAA-CC | AAG TGC ATC CCC AAC GTC TG |
| minGAA-CA | AAG TGC ATC CAC AAC GTC TG |
| minGAA-CT | AAG TGC ATC CTC AAC GTC TG |
| minGAA-CG | AAG TGC ATC CGC AAC GTC TG |
| minGAA-77 | GTG CAT CAC CAA CGT C |
| *cis*-minGAA- type-1 | GGA TCA CCC GCG AAG CG [2**´**-*o*-methyl G] rArAG GAT CC |
| minGAA-s-type-2 | GAT CAC CCG C |
| *cis*-minGAA- type-1-dA19 | GGA TCA CCC GCG AAG CG [2**´**-*o*-methyl G] ArAG GAT CC |
| *cis*-minGAA- type-1-dA20 | GGA TCA CCC GCG AAG CG [2**´**-*o*-methyl G] rAAG GAT CC |
|  |  |
| **Substrates used in this study (5´ to 3´)** | |
| sub-N10 | ATC TGA ACT AGG CAG ACG TTrG rArAG GAT GCA CTT-biotin |
| sub-N3 | AGA CGG ATC AAG TCT ACrG rArArG GAG GTA CTT-biotin |
| sub-rArAG | ATC TGA ACT AGG CAG ACG TTrG rArAG GAT GCA CTT-FAM |
| sub-rCrAG | ATC TGA ACT AGG CAG ACG TTrG rCrAG GAT GCA CTT-FAM |
| sub-rArCG | ATC TGA ACT AGG CAG ACG TTrG rArCG GAT GCA CTT-FAM |
| sub-rArAC | ATC TGA ACT AGG CAG ACG TTrG rArAC GAT GCA CTT-FAM |
| sub-allRNA | rAUrC UrGrA rArCU rArGrG rCrArG rArCrG UUrG rArArG rGrAU rGrCrA rCUU-FAM |
| sub-singleRNA | ATC TGA ACT AGG CAG ACG TTrG AAG GAT GCA CTT-FAM |
| sub-rArArAG | ATC TGA ACT AGG CAG ACG TTrA rArAG GAT GCA CTT-FAM |
| sub-rCrArAG | ATC TGA ACT AGG CAG ACG TTrC rArAG GAT GCA CTT-FAM |
| sub-UrArAG | ATC TGA ACT AGG CAG ACG TTU rArAG GAT GCA CTT-FAM |
| sub-type-2 | GCG [2**´**-*o*-methyl G] rArA GGA TC |

**Table S2: Data collection and refinement statistics in crystallography**

|  | molecules | |
| --- | --- | --- |
| Crystallographic data^a^ | Type 1 | Type 2 |
| Space group | C121 | P222 |
| Unit cell |  |  |
| a/b/c (Å) | 68.10/50.38/51.38 | 38.86/81.88/81.78 |
| α/β/γ (°) | 90.0/107.0/90.0 | 90.0/90.0/90.0 |
| Wavelength (Å) | 1.08 | 1.08 |
| Resolution range (outer shell) (Å) | 49.16–2.85  (3.02–2.85) | 40.94–3.04  (3.22–3.04) |
| Total reflections | 13,021 | 33,787 |
| Unique reflections | 3,855 | 5,372 |
| Completeness (outer shell) (%) | 92.6 (85.7) | 99.4 (97.1) |
| R_merge_ (outer shell) (%) | 4.2 (62.6) | 4.6 (45.0) |
| Average I/σ(I) (outer shell) | 9.4 (1.1) | 13.9 (1.9) |
| Refinement |  |  |
| R_work_/R_free_ (%) | 24.3/32.9 | 21.0/29.7 |
| RMSD from ideal values |  |  |
| Bond length (Å) | 0.005 | 0.005 |
| Bond angle (°) | 1.2 | 1.3 |
| Average B-factors (Å^2^) (Number of atoms) |  |  |
| Nucleic acid | 113.0 (1091) | 104.3 (1658) |
| Metal ion | 128.5 (4) | 135.1 (4) |
| Water | 98.0 (6) | 88.5 (23) |

**Table S3: Structural statistics for NMR structure determination**

| Structural restraints^a^ |  |
| --- | --- |
| NOE | 530 |
| Intra-nucleotide NOEs^b^ | 168 |
| Sequential NOEs (\|i–j\|=1) | 289 |
| Long-range NOEs (\|i–j\|≧2) | 73 |
| Theoretical^c^ | 121 |
| Distance (Base pairing) | 58 |
| (Zn^2+^ coordination) | 1 |
| Dihedral (B-DNA backbone) | 52 |
| Planarity (Base paring) | 9 |
| (Zn^2+^ coordination) | 1 |
| Total | 651 |
| Characteristics of 20 selected structures |  |
| RMSD from restraints |  |
| Distances (Å) | 0.0066 ± 0.0004 |
| Torsion angles (degrees) | 0.09 ± 0.01 |
| van der Waals energy (kcal/mol)^d^ | 0.7 ± 0.3 |
| RMSD from the ideal geometry | |
| Bond lengths (Å) | 0.0024 ± 0.0001 |
| Bond angles (degrees) | 0.435 ± 0.006 |
| Improper angles (degrees) | 0.222 ± 0.002 |
| Average RMSD to mean structure (Å)^e^ |  |
| Whole molecule | 1.25 ± 0.33 |
| Core region^f^ | 0.48 ± 0.13 |

^a^Restraints used in the final calculation (see Supplementary Materials).

^b^NOEs between base and sugar protons.

^c^See Supplementary Methods in detail.

^d^Value for the repel function in the CNS package.

^e^Nonhydrogen atoms were selected.

^f^Value for T4–C9/G17–A23 except for the flexible rA19.


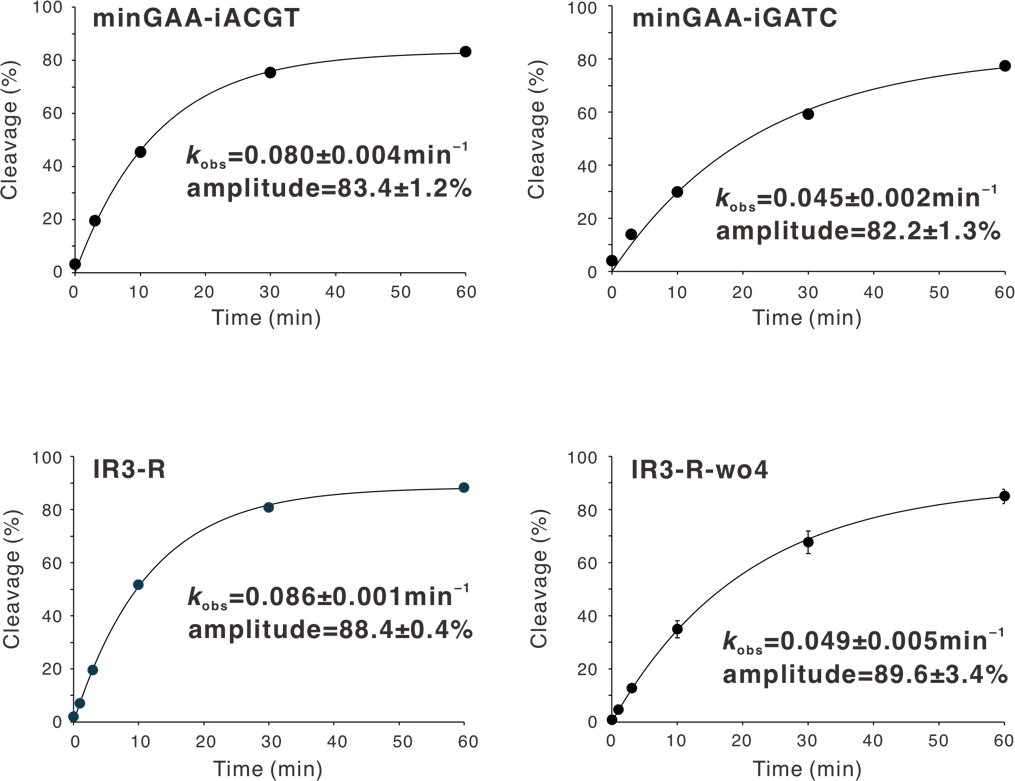


Figure S1: Kinetic experiments for intermediate molecules during minimization of DNAzyme.


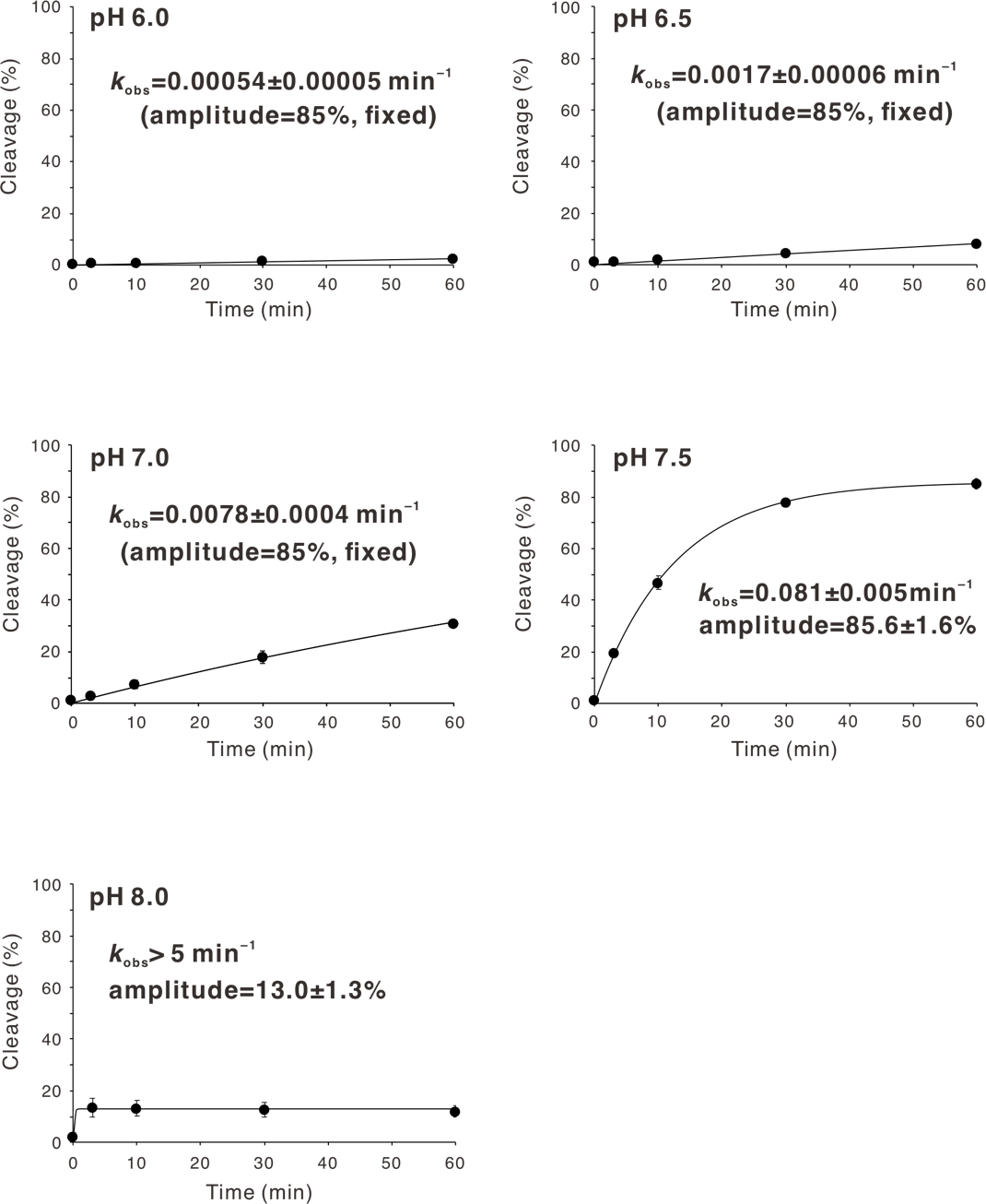


Figure S2: Kinetic experiments for minGAA (minGAA-s) at different pH.


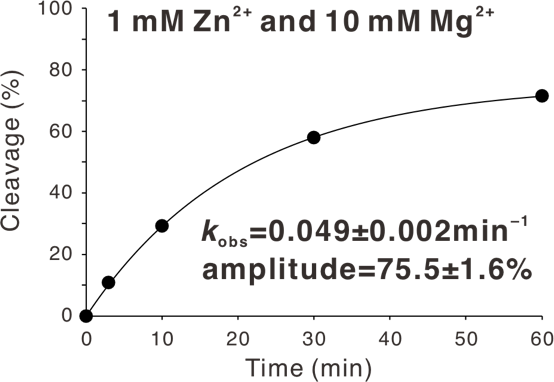


Figure S3: Kinetic experiments for minGAA (minGAA-s) in the presence of Zn^2+^ and Mg^2+^ ions.


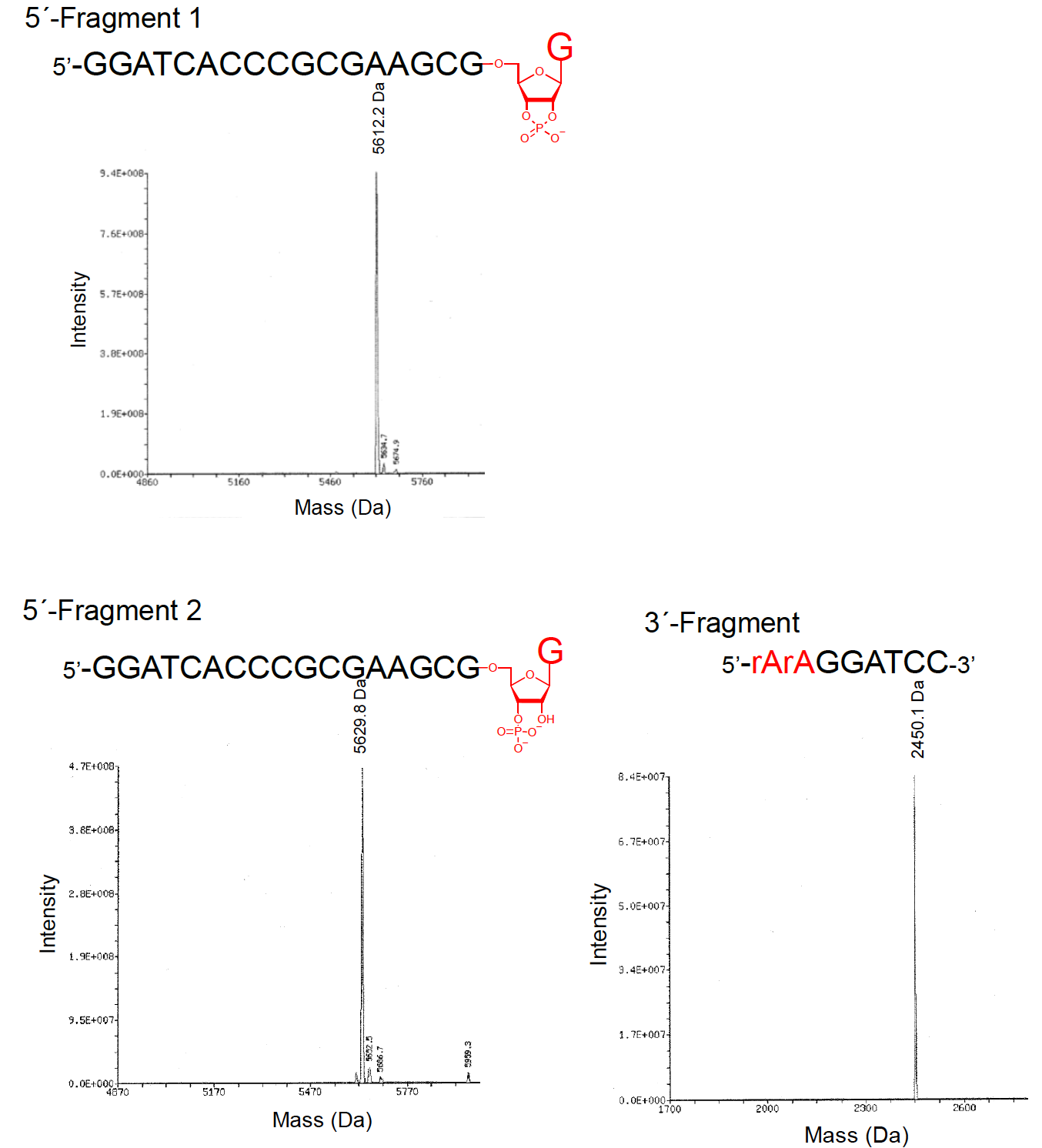


Figure S4: LC-MS of the fragments of the *cis*-type hairpin minGAA after cleavage. In the sequences shown, the RNA moieties are shown in red.


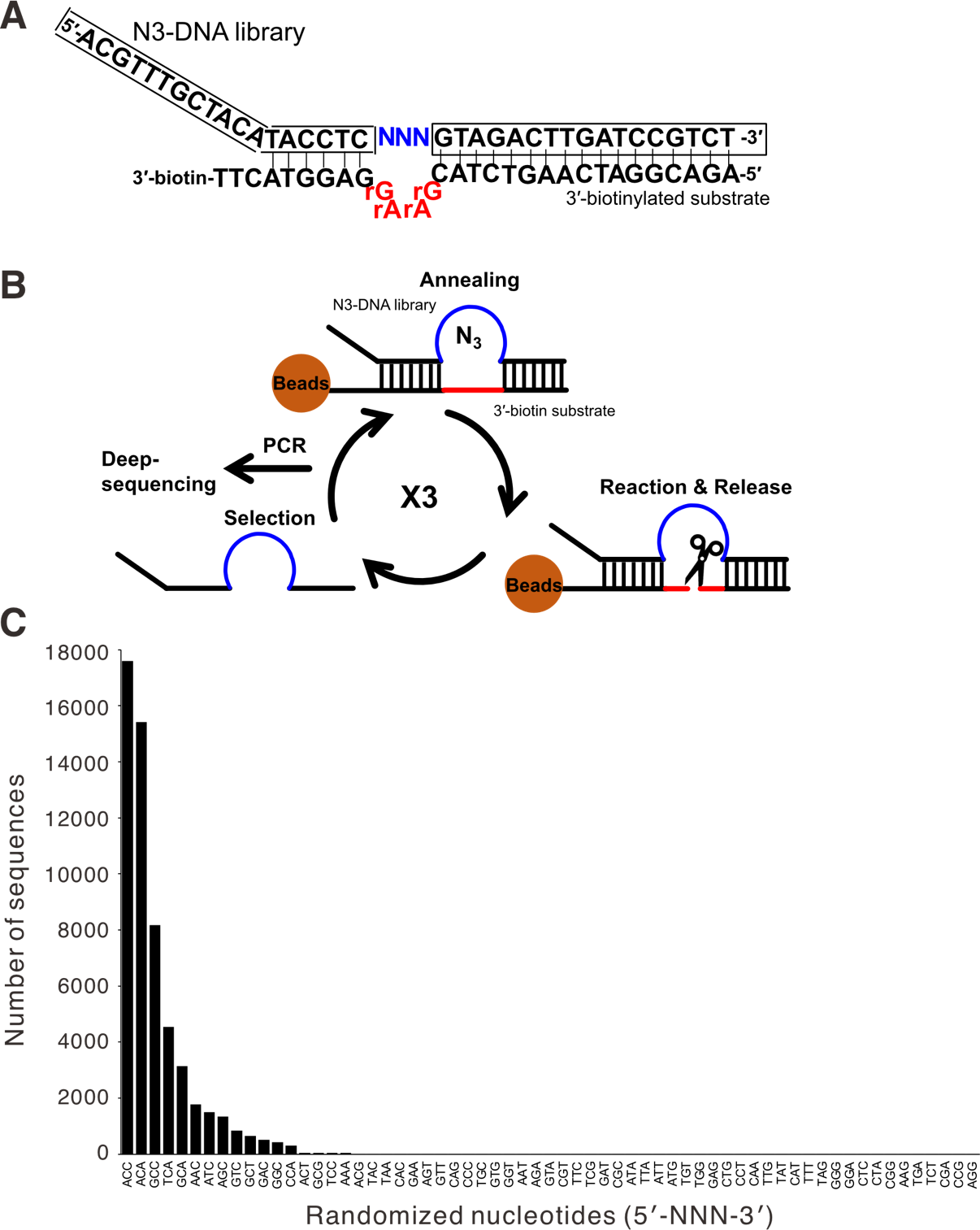


Figure S5: Optimization of minGAA catalytic sequence by *in vitro* selection. (A) The N3-DNA library and 3′-biotinylated substrate. The arm sequences, corresponding to the primers for amplification (including complementary sequence), are enclosed by the dotted line. The catalytic core region and the core substrate region are shown in blue and red, respectively. (B) The *in vitro* selection procedure. In each cycle, the molecules that exhibited cleavage activity were released into the solution. (C) Number of enriched catalytic core sequences after *in vitro* selection. DNAzyme that has more enriched sequence appears to be released into the solution by cutting substate more efficiently.


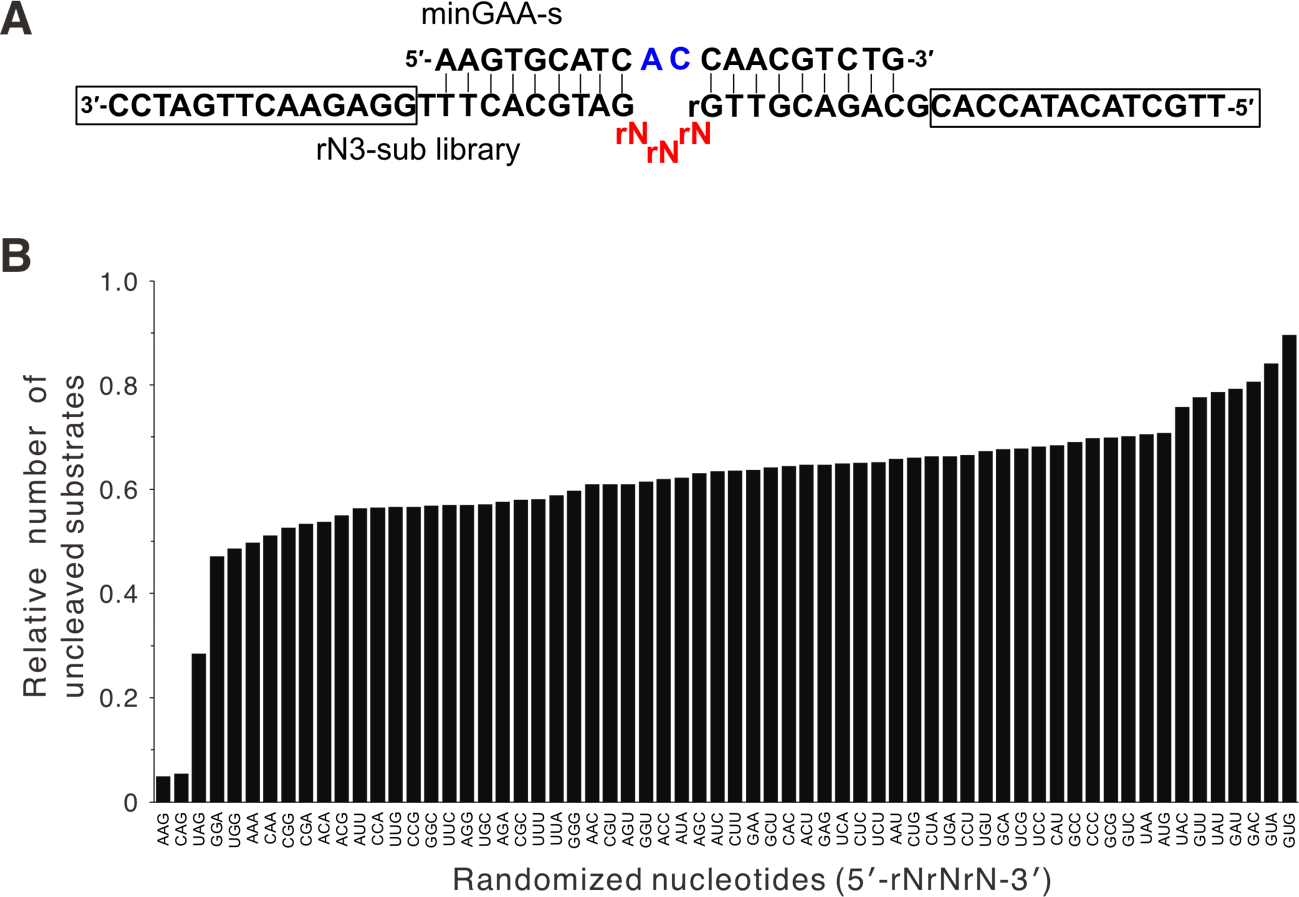


Figure S6: Preferred substrate core sequence of minGAA analyzed by using a random- nucleotide library. (A) The minGAA-s and N3-RNA library with 3-nt randomized region for substrate. The arm sequences, corresponding to the primers for the PCR amplification, are enclosed by the dotted line. The catalytic core region and the core substrate region are shown in blue and red, respectively. (B) The relative number of the substrate that was not cleaved by minGAA. The substrate library was cleaved by minGAA under the single turnover condition in the buffer with or without 1 mM Zn^2+^. The remaining uncleaved molecules were amplified and was analyzed by the next-generation sequencer. Numbers of the sequences in the presence of Zn^2+^ were divided by those in the absence of Zn^2+^.


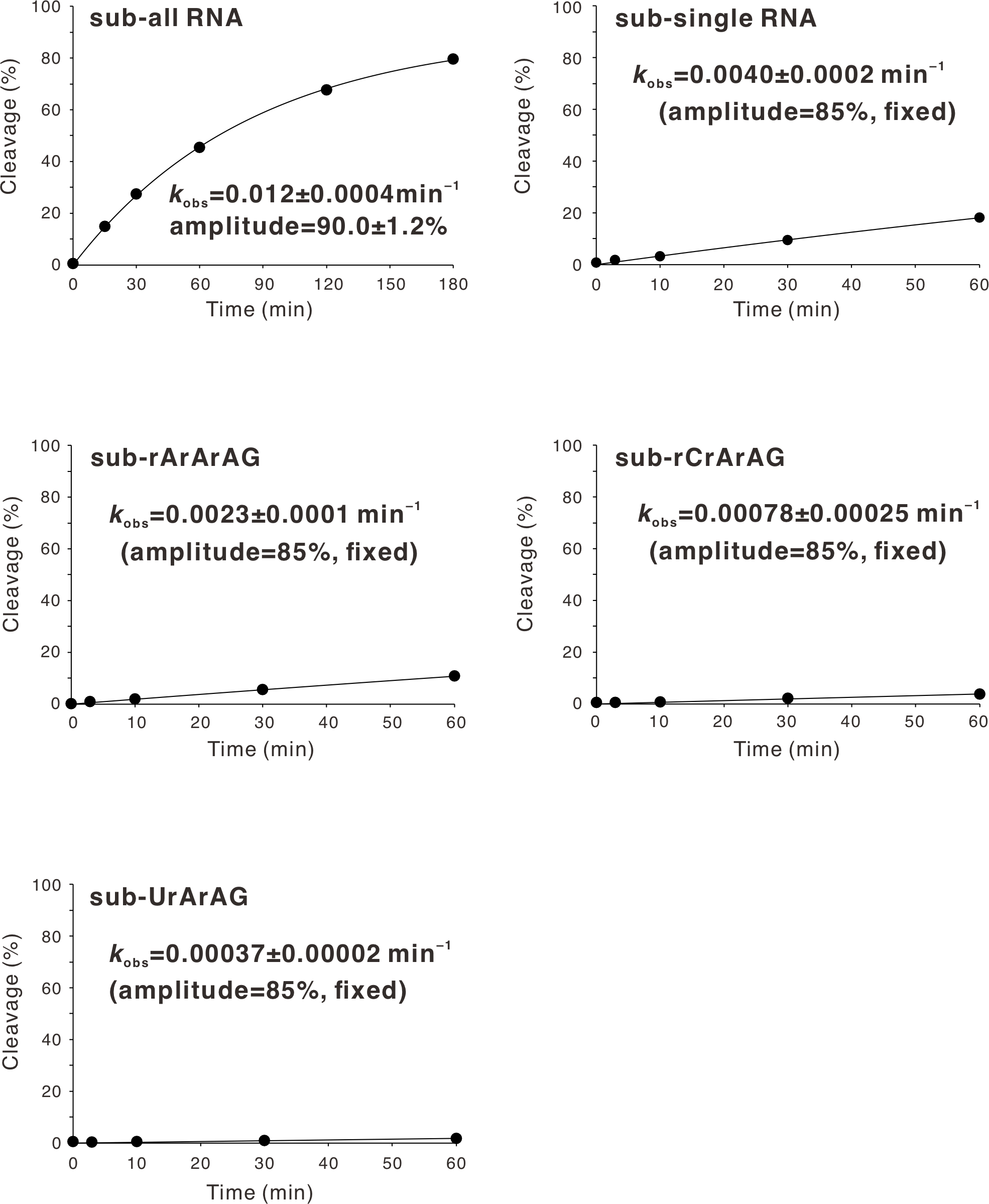


Figure S7: Kinetic experiments for minGAA (minGAA-s) with modified substrates.


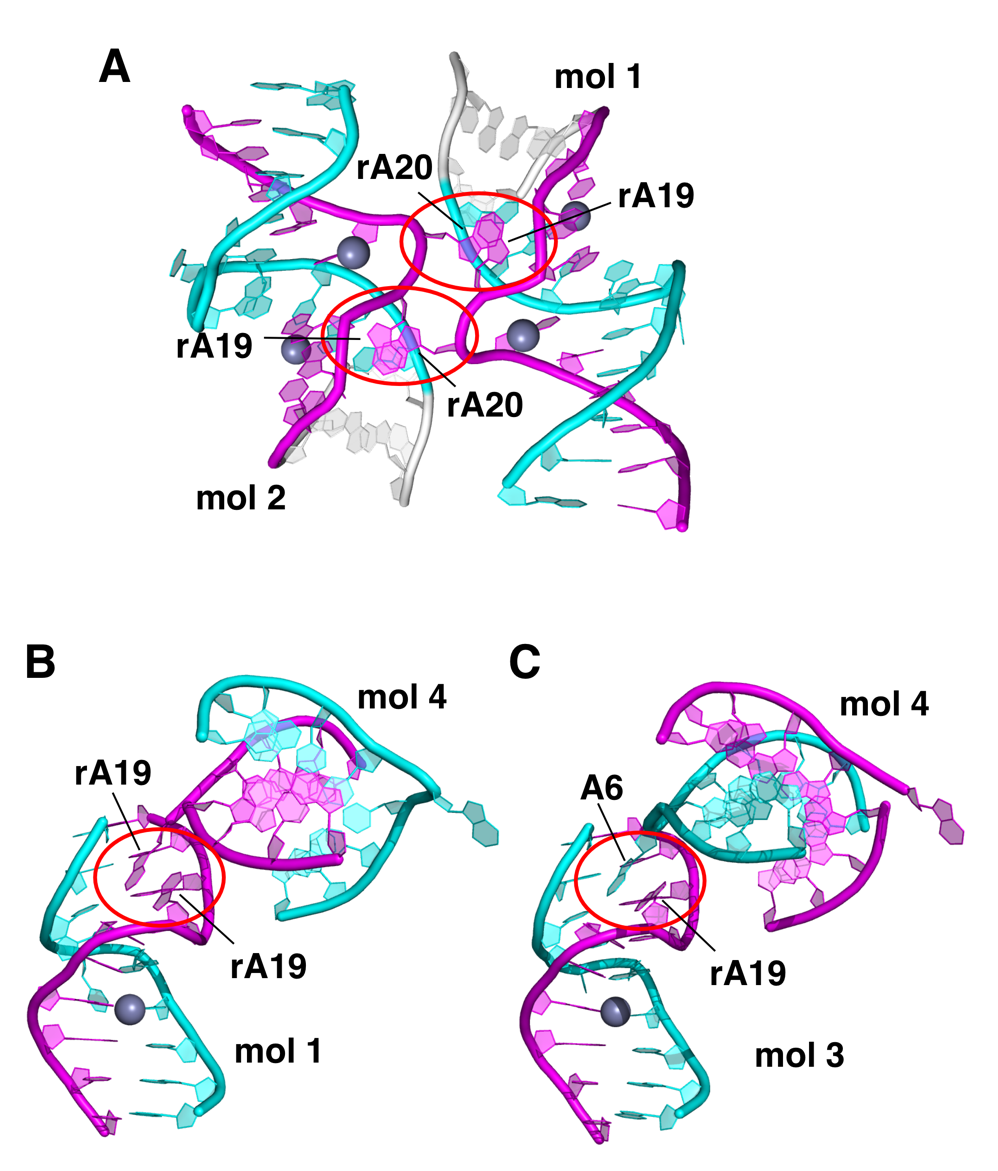


Figure S8: Base stacking observed between adjacent molecules in the crystal lattice observed for type 1 (A) and type 2 (B,C) molecules. Red circles highlight the stacking regions.


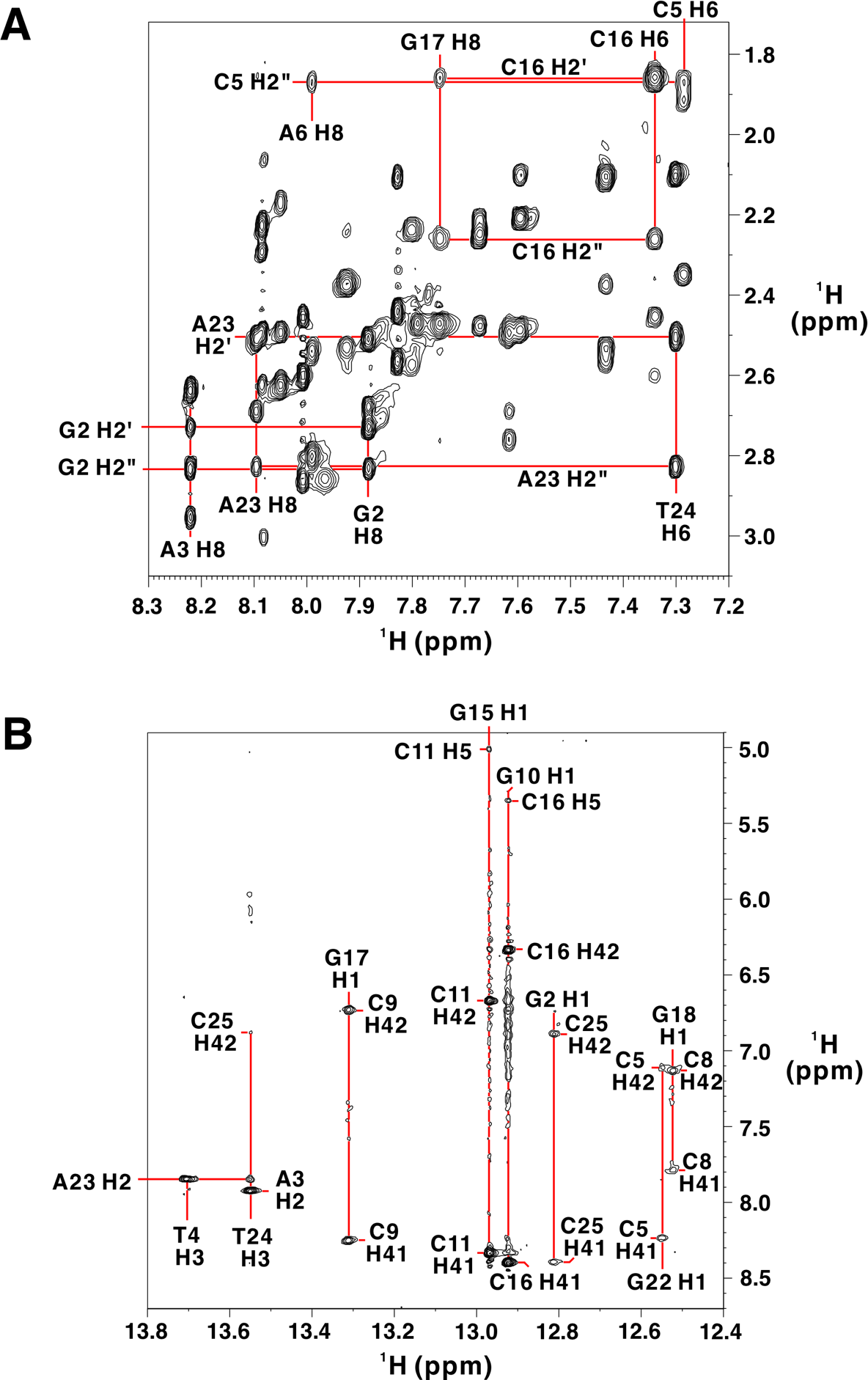


(continued)


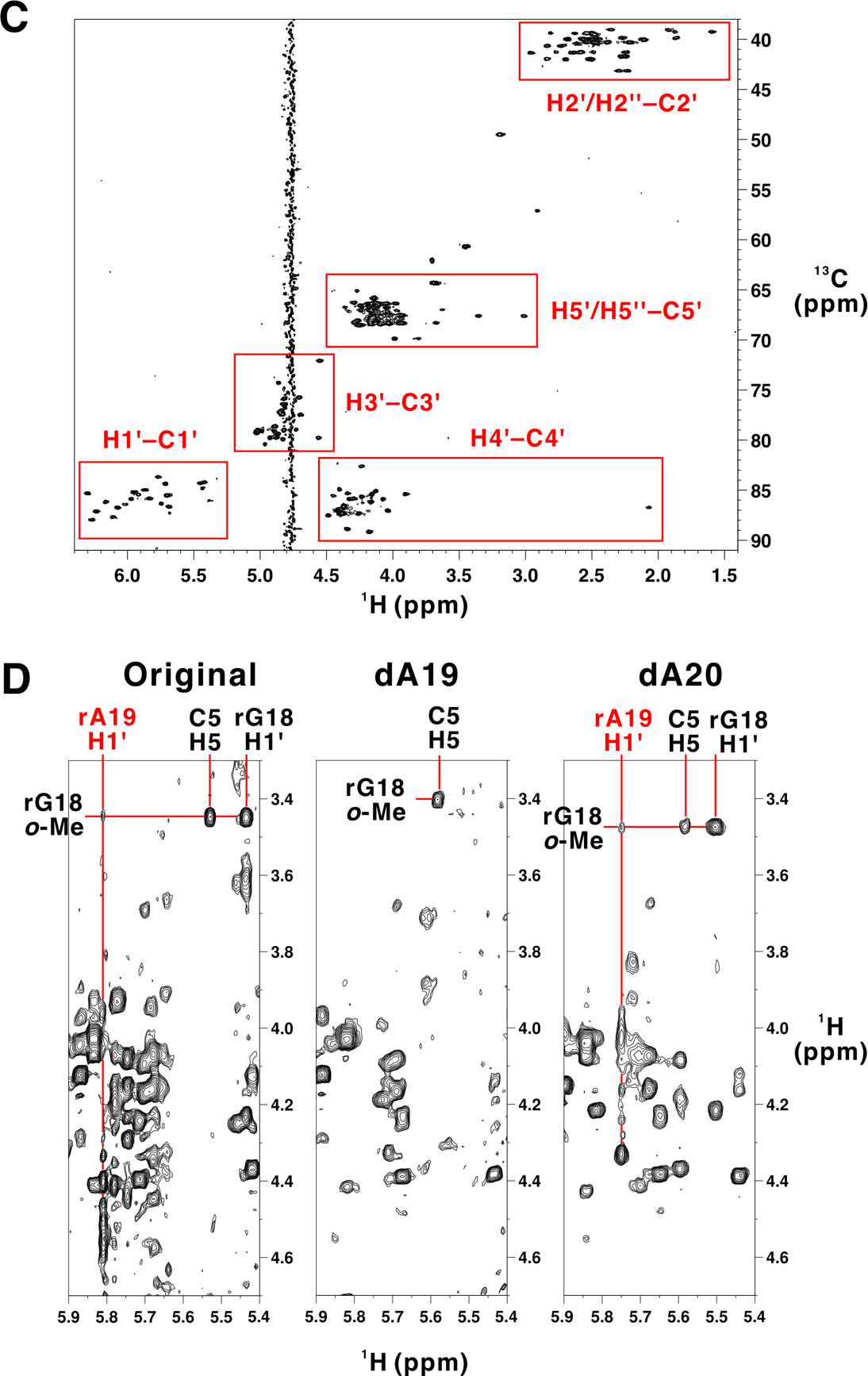


Figure S9: NMR spectra for resonance assignments. (A) the nuclear Overhauser effects (NOEs) between adjacent nucleotides, (B) NOEs showing base pairing, (C) ^1^H–^13^C coherences, and (D) RNA/DNA mutations at rA19 and rA20.


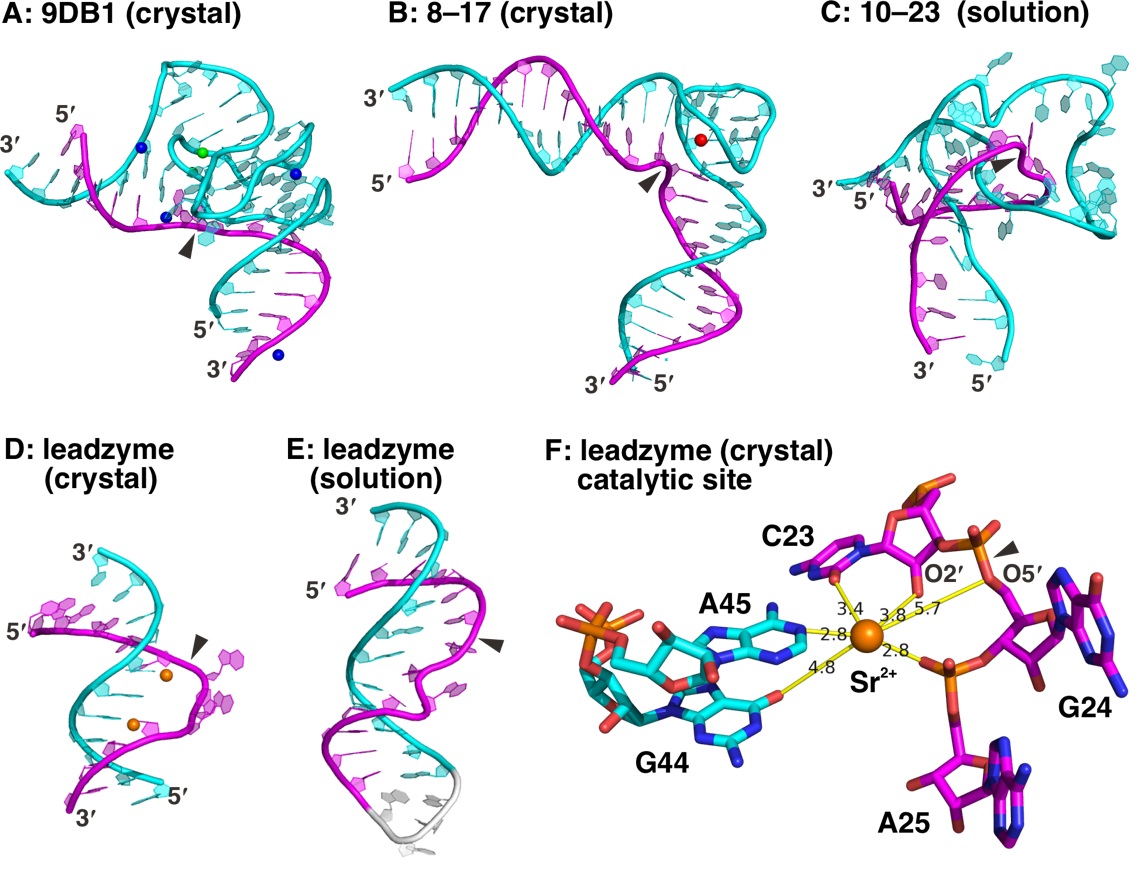


Figure S10: Crystal and solution structures of related DNAzymes and ribozymes. Shown are structures of (A) 9DB1, (B) 8–17, and (C) 10–23 DNAzymes and leadzyme in (D) crystal and (E) solution. In (F), catalytic site of leadzyme in crystal is viewed in detail, where distances between atoms are shown in Å. Catalytic and substrate strands or moieties are shown in cyan and magenta, respectively. The cleavage sites are indicated by arrowheads. Metal ions are Co^2+^ (blue) and Mg^2+^ (green) in (A), Pb^2+^ (red) in (B), and Sr^2+^ (orange) in (D).


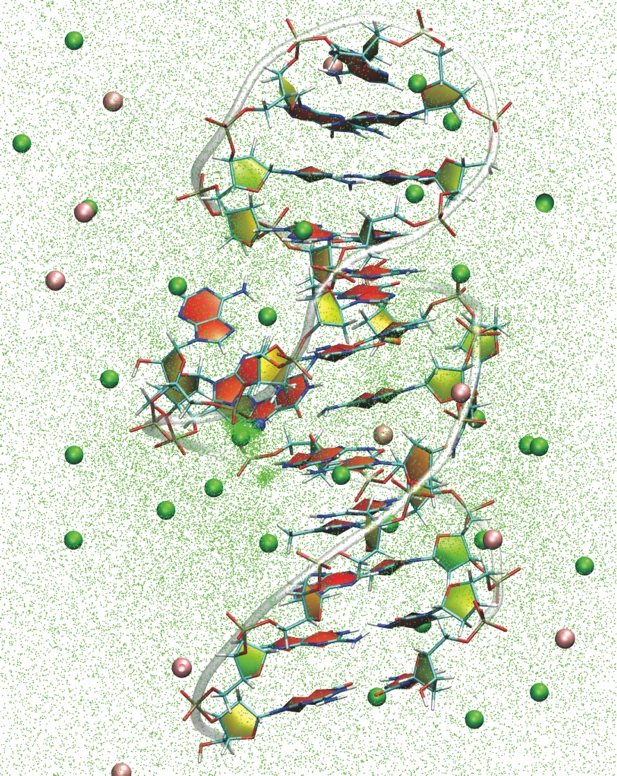


Figure S11: The distribution of Na⁺ ions during the 2-μs MD simulation of the preliminary Zn²⁺-free NMR structure of minGAA (without Zn²⁺). Green dots represent the time-integrated distribution of Na⁺ ions throughout the simulation, while green and red spheres indicate the positions of Na^+^ and Cl^-^ ions, respectively, in the snapshot after equilibration. Figure was created by VMD^13^.
